# Shared decision making in primary malignant bone tumour surgery around the knee in children and young adults: protocol for a prospective study

**DOI:** 10.1186/s13018-024-05192-y

**Published:** 2024-11-02

**Authors:** Kiki J Blom, Willem P Bekkering, Marta Fiocco, Michiel AJ van de Sande, Hendrik WB Schreuder, Lizz van der Heijden, Paul C Jutte, Lianne M Haveman, Johannes HM Merks, Jos AM Bramer

**Affiliations:** 1 Department of Orthopedic Surgery and Sports Medicine, Amsterdam Movement Sciences, Amsterdam University Medical Centers, Amsterdam, the Netherlands; 2 Princess Maxima Centre for Paediatric Oncology, Utrecht, The Netherlands; 3 Mathematical Institute, Leiden University, Leiden, the Netherlands; 4 Department of Biomedical Data Sciences, Medical Statistics Section, Leiden University Medical Center, Leiden, the Netherlands; 5 Department of Orthopaedic Surgery, Leiden University Medical Centre, Leiden, the Netherlands; 6 Department of Orthopaedic Surgery, Radboud University Medical Centre Nijmegen, Nijmegen, the Netherlands; 7 Department of Orthopaedic Surgery, University Medical Centre Groningen, Groningen, the Netherlands; 8 Division of Imaging and Oncology, University Medical Centre Utrecht, Utrecht University, Utrecht, the Netherlands; 9 Cancer Center Amsterdam, Amsterdam University Medical Centers, Amsterdam, the Netherlands

**Keywords:** Bone tumour surgery, Children and adolescents, Decisional conflict, Decisional regret, Ewing’s sarcoma, Oncology, Osteosarcoma, Quality of life, Shared decision making

## Abstract

**Background:**

Children and young adults needing surgery for a primary malignant bone tumour around the knee face a difficult, life-changing decision. A previous study showed that this population wants to be involved more in the decision-making process and that more involvement leads to less decisional stress and regret. Therefore, a well-designed and standardized decision-making process based on the principles of shared decision-making needs to be designed, implemented, and evaluated.

**Methods:**

We developed a shared decision-making (SDM) model for this patient population, including an online decision aid (DA). This model has been implemented in the standard care of patients with a primary malignant bone tumour around the knee. Following implementation, we will analyse its effect on the decision-making process and the impact on patient experiences using questionnaires and interviews. Moreover, potential areas for improvement will be identified.

**Discussion:**

Given the importance of involving patients and parents in surgical decision-making, particularly in life-changing surgery such as malignant bone tumour surgery, and given the lack of SDM models applicable for this purpose, we want to share our model with the international community, including our study protocol for evaluating and optimising the model. This study will generate valuable knowledge to facilitate the optimisation of current patient care for local treatment. The sharing of our implementation and study protocol can serve as an example for other centres interested in implementing SDM methods in an era characterized by more empowered patients and parents who desire autonomy and reliable and realistic information.

**Supplementary Information:**

The online version contains supplementary material available at 10.1186/s13018-024-05192-y.

## Background

Primary malignant bone tumours typically occur in children, adolescents, and young adults [1]. The majority of these tumours are osteosarcoma or Ewing’s sarcoma located around the knee [2]. These tumours exert a significant impact on the patient’s life. Treatment protocols typically involve a multimodal approach, including preoperative chemotherapy, tumour resection, and postoperative chemotherapy. In Ewing’s sarcoma, radiotherapy can be added, before or after surgery, or used as a single local treatment [3, 4]. Advances in chemotherapy regimens have significantly improved survival rates, transforming the treatment landscape and shifting the focus toward optimizing surgical outcomes.

In a curative setting, the entire tumour must be removed with at least a margin of healthy tissue around it. In limb-saving surgery, resection is followed by reconstruction of the remaining defect. In the late 70’s around 80% of the patients underwent an amputation; nowadays due to response to neoadjuvant chemotherapy, improved imaging technologies, and advances in surgical interventions this shifted to a limb-saving surgery for 85% of the patients [5, 6]. There are four different surgical options for tumours around the knee: amputation, rotationplasty, resection with prosthetic or biological reconstruction. Amputation involves the partial or complete removal of the affected limb. Rotationplasty entails removing the tumour-bearing segment while preserving and rotating the lower leg and foot to reattach it as a functional knee joint. Endoprosthetic replacement involves substituting the resected bone with a custom metal implant. Biological reconstruction uses donor bone (allografts), the patient’s own bone (autografts), or a combination (allograft-prosthesis composite) to rebuild the defect [5, 7, 8]. Each of these options represents a life-altering surgical intervention, with varying implications for surgical margins, rehabilitation, aesthetics, and functionality.

The literature reports no significant differences in functionality or quality of life (QoL) between limb-saving and ablative surgery [9–15]. However, compared with other childhood cancer survivors, these patients consistently report remarkably lower QoL scores, especially concerning physical functioning in everyday life, participation in sports, and self-esteem [16–18].

Selecting the optimal surgical approach for each patient within a limited timeframe, ranging from 9 to 18 weeks, presents a significant challenge. This critical decision must be made during preoperative chemotherapy, a highly confusing and distressing period, especially for children who may be severely ill. While oncological safety and surgical factors are paramount to surgery, it is essential to consider other aspects such as functional, and cosmetic factors, alongside potential complications.

Additionally, factors such as the patient’s age and remaining limb growth potential, future aspirations, activities, preferences, and expectations, as well as those of their families, must be taken into consideration [19]. Currently, there are no established guidelines for determining which surgery to choose in these complex scenarios [5–7]. The surgeon’s opinion and experience play an important role, both consciously and subconsciously [7, 20].

Physicians hold both ethical and legal responsibilities to provide patients with comprehensive information regarding available treatment options, potential risks and consequences, thereby actively facilitate patient participation in decision-making [21, 22]. In recent years, shared decision-making (SDM) has emerged as the preferred model for improving patients’ knowledge and understanding of options, reducing emotional distress and decisional regret, and aligning decisions with the patients’ and families’ values [23–26].

A previous study demonstrated that patients with a primary malignant bone tumour around the knee, along with their parents, express a desire to be involved in the decision-making process. Moreover, increased involvement correlates with reduced decisional stress, which in turn correlates with less decisional regret [27]. Hence, there is a pressing need for a well-designed and standardized decision-making process based on the principles of SDM. To address this need, we have developed an SDM model tailored to this patient population that incorporates an online decision aid. This model has been integrated into our standard-of-care procedures. As part of its implementation, we will assess the impact of SDM on the surgical decision-making process through questionnaires and interviews with young bone sarcoma patients. This analysis will enable us to continuously refine the process as part of our standard care protocol. Recognizing the significance of a robust SDM process for this vulnerable population undergoing life-changing surgery, and acknowledging the absence of existing SDM models, we aim to share our model with the international community, along with our study protocol for evaluating and enhancing the model.

## Methods

This paper is structured in two parts. Part I details the development of the decision aid and the creation and implementation of the SDM model. Part II outlines the study design, aimed at assessing the impact of the implemented model and identifying opportunities for its improvement.

### Part I - DEVELOPMENT of the SDM model

#### Decision aid

We developed a digital decision aid based on insights gathered from a survey conducted with patients, parents, and healthcare professionals to understand their needs and preferences. The survey findings underscore the desire of patients with primary malignant bone tumours and their parents to actively participate in the decision-making process. Furthermore, increased involvement correlates with reduced decisional stress, which in turn correlates with less decisional regret [27]. Participants also reported a lack of supportive visual materials, such as videos and photographs, while seeking information about surgical options. Topics such as rehabilitation (20–29%), the post-surgery situation (14–19%), impact on daily life (11–19%), sports (15–20%), and cosmetic outcomes (14–17%) were reported as inadequately covered (unpublished results). To ensure the active involvement of both patients and parents, it was imperative to provide them with comprehensive information. Consequently, the decision aid was designed as a comprehensive tool, facilitating informed decision-making and empowering both patients and their parents throughout the decision-making process.

The decision aid was developed to deliver age-appropriate information to young patients and their parents, addressing the gap in available information and catering to their specific informational needs. Throughout its development, patients actively provided feedback, enabling ongoing improvement and refinement of the tool to better meet their needs. Its primary aim is to facilitate an informed decision-making process. Currently available in Dutch (https://www.amc.nl/keuzehulpbottumor), efforts are underway to translate the decision aid into other languages.

The decision aid comprises five key components: an informative text, animated films illustrating the four surgical procedures, realistic photographs depicting potential outcomes, an “option grid,” and preference questions.

The following four surgical options are discussed in the decision aid (Appendix 1):

1. Resection, followed by reconstruction with an internal prosthesis.
2. Resection, followed by biological reconstruction.
3. Rotationplasty [28].
4. Amputation.

The choice of surgical options for each patient is contingent upon the location of the tumour and its involvement with adjacent tissues. By clicking on each surgical option, the informative text and animated film will be shown. While information on all surgical options is accessible in the decision aid, patients have the freedom to choose which specific option they wish to explore. This approach prevents patients and parents from being inundated with unsolicited explanations of surgical alternatives about which they may not be interested.

##### Informative text

The digital decision aid commences with a brief introductory overview outlining the target patient population for which the decision aid is intended, and the topics and components to be addressed. A concise explanation follows, explaining the two most prevalent malignant bone tumours in children and young adults - osteosarcoma and Ewing sarcoma - alongside their respective treatments, including the necessity of resection. The subsequent segment delves into the surgical aspects of treatment, presenting four distinct surgical options for consideration. Decision aid users have the autonomy to select the option(s) they wish to explore further. The system is designed to shield patients from exposure to unwanted information and prevent information overload. Upon clicking on the relevant option(s), information becomes visible. The topics discussed include surgical procedures, rehabilitation processes, potential complications, and sports options. Each surgical option is concluded with a comprehensive list outlining its potential advantages and disadvantages.

##### Animated films

**Fig. 1:**
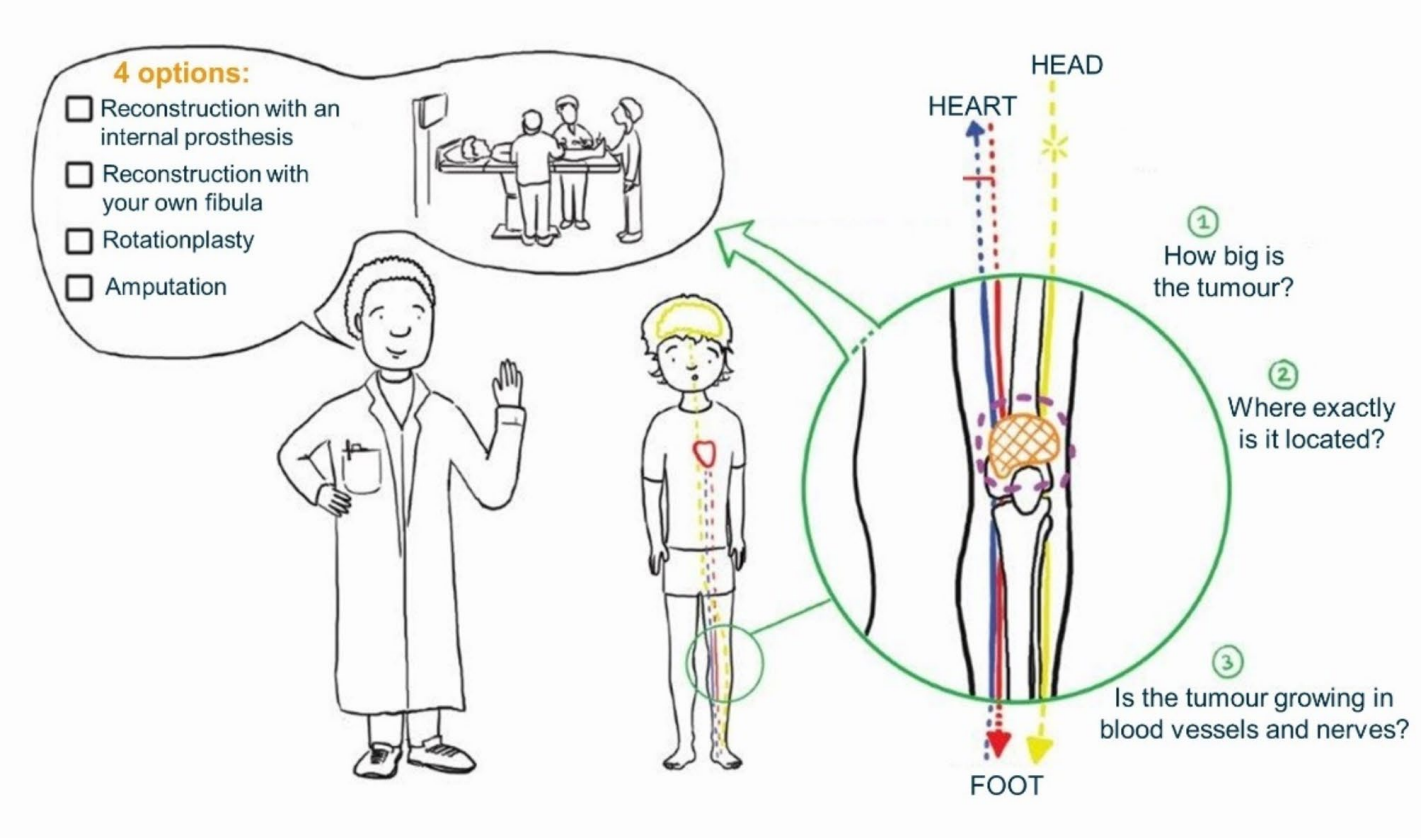
Image of the introductory film

A total of five animated films were created featuring an illustrated character designed to be engaging and comprehensible for individuals of all ages. These films include an introductory film and four films depicting the four surgical techniques. The introductory film outlines the necessity of tumor removal with a margin, introduces the four available surgical options, and discusses the factors influencing the selection of surgery for each patient (Fig. 1). The surgical option films illustrate the process of tumour resection and leg reconstruction (Appendix 1). Additionally, they provide insights into posttreatment care, rehabilitation following surgery, and future opportunities for sports involvement. The animated films are suitable for children as young as 8 years old but remain engaging and informative for teenagers and young adult patients.

##### Realistic photographs

the decision aid incorporates photos of (former) patients to illustrate potential post-surgery outcomes. This offers young patients and their parents a realistic depiction of what to expect regarding the cosmetic consequences of surgical options.

##### Option grid

The option grid provides a concise, easily digestible table summarizing key information for each surgery. It includes details such as cosmetic outcomes, rehabilitation requirements, post-rehabilitation walking ability, sports abilities, oncological safety, short- and long-term complication risks, and the potential need for future surgeries. This table enables patients and their parents to quickly compare the similarities and differences among surgical options.

##### Preference questions

Towards the end of the decision aid, knowledge-based questions assess the user’s understanding of the essential information. Finally, preference questions prompt users to assign values to presented statements, aiding in the recognition of personal preferences. If desired, the answers to these questions can be used in discussions with the attending orthopaedic surgeon.

## Development and implementation of the shared decision-making model

**Fig. 2:**
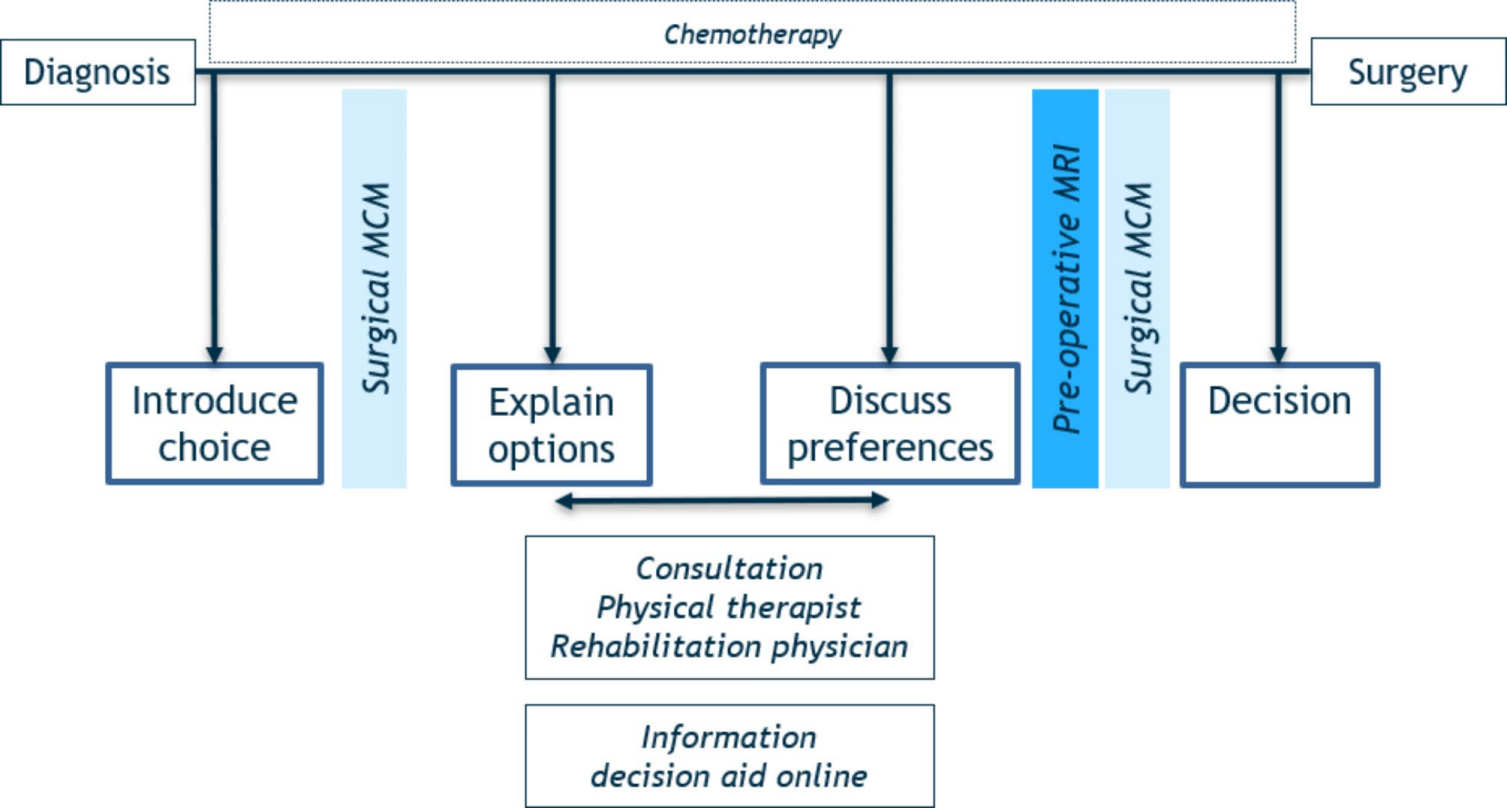
Implementation of the SDM model into the treatment schedule for primary malignant bone tumours. *MCM: Multidisciplinary consultation meeting*,* MRI: Magnetic resonance imaging*

In recent years, several models have been formulated for implementing SDM in practical settings. Within our framework, the model proposed by Stiggelbout et al. seemed most suitable for integrating SDM into the decision-making process for the identified target population [23]. Stiggelbout’s model delineates four sequential steps. We implemented these four steps in the surgical decision-making process for the treatment of primary malignant bone tumours in children and young adults (Fig. 2). Consultations occur at sequential intervals prior to surgery, typically spaced between 1 and 3 weeks apart. The duration from diagnosis to surgery depends on the type of tumour: 11 weeks for osteosarcoma patients and 20 weeks for Ewing sarcoma patients. Whenever feasible, consultations are aligned with the patient’s hospitalisation for chemotherapy, to minimize additional hospital visits (Appendix 2). These consultations involve the patient, parents, orthopaedic surgeon, and nurse practitioner.

Consultation 1: Within 2 weeks of diagnosis, either the orthopaedic surgeon or the oncologist initiates a conversation with the patient and parents regarding the need to make a decision. They explain that surgery is a necessary component of the treatment plan. This conversation emphasizes the invaluable input of both the patient and their parents. Additionally, they will inform that a more detailed conversation about surgical options will take place in the upcoming weeks.

Consultation 2: Approximately 1–2 weeks after this conversation, the patient, parents, and orthopaedic surgeon convene for a second scheduled SDM meeting. In this meeting, the orthopaedic surgeon explains in a neutral way the options available for this patient, detailing the associated advantages and disadvantages, i.e., their benefits and harms. At the conclusion of this second consultation, the digital decision aid is offered, providing additional information about relevant surgical options and allowing the patient and parents to view animated films at their convenience in preparation for the third consultation with the orthopaedic surgeon to discuss the patient’s preferences.

During the interim period, the patient and their parents will meet with the physical therapist and rehabilitation physician, who will provide further insights into the functional consequences of the surgical options and the accompanying rehabilitation processes. They also assess the need for any supplementary educational materials, such as videos or contact with peers.

Consultation 3: Following discussions with the physical therapist and rehabilitation physician, the patient and parents engage in a third conversation with the orthopaedic surgeon to ask questions and discuss preferences. While no decisions are finalised during this session, both the patient and the orthopaedic surgeon’s preferences are thoroughly discussed.

Consultation 4: The final decision-making session occurs shortly after the preoperative MRI. During this meeting, all medical information and insights are integrated with the patient’s preferences to collaboratively reach a decision. Once a decision is made, the orthopaedic surgeon outlines the forthcoming steps and what the patient can expect in the ensuing weeks.

Since September 2021, the SDM model has been implemented as a standard of care in the Amsterdam University Medical Centre (UMC) and Princess Máxima Centre for Paediatric Oncology and in subsequent years in the other three Dutch orthopaedic oncological centres (Leiden UMC, Radboud UMC, UMC Groningen).

### Part II - evaluation of the SDM model

#### Objectives

Here, we present the intended outcomes of our research, aiming to systematically evaluate the impact of integrating the model into standard care.

Primary outcome: perceived level of involvement.

Secondary outcomes: decisional conflict, decisional regret, daily functioning, and quality of life.

Methodology for optimizing the SDM model: identifying potential avenues for enhancing the implemented model.

### Study design

This is a prospective nationwide multicentre study conducted at the Princess Máxima Centre and all four Dutch orthopaedic oncological centres. Patient recruitment started in October 2021. It is anticipated that the inclusion of patients will be completed by early 2025.

### Patients

#### Inclusion criteria

To be included, a patient must fulfil all of the following criteria: (1) age less than 26 years at diagnosis; (2) primary malignant bone tumour: osteosarcoma or Ewing sarcoma; (3) tumour localized around the knee joint, i.e. in the proximal tibia, fibula or distal femur; and (4) at least two of the four surgical treatment options should be technically feasible for the patient, as determined by the orthopaedic surgeon, i.e. amputation, rotationplasty, resection with an internal prosthesis and/or a biological reconstruction. Patients and parents or legal guardians (if applicable) must have sufficient understanding of the Dutch language to complete the questionnaires. For the interviews, a certified translator can be used if necessary.

### Sample size

The primary outcome measure of this study is the perceived level of involvement in the decision process. A minimum sample size of 24 patients is required to detect a difference between the perceived level of involvement measured during the 2015–2016 baseline survey and this study, with an effect size of 0.608 and 80% power with a significance level (alpha) of 5%.

### Informed consent

Patient inclusion is performed by the attending orthopaedic surgeon. If a patient meets all the inclusion criteria, the treating orthopaedic surgeon will inform the patient and/or parents/legal guardians (if applicable) about the study and invite them to participate. Only patients and/or parents who provide signed informed consent will be included in the study.

### Patient characteristics

At baseline, sociodemographic data will be collected from participating patients, including their sex, date of birth, level of education, and ethnicity. In addition, information about the patient’s family will be gathered through a questionnaire completed by the parent or guardian. This includes details about the parent’s sex, age, ethnicity, marital status, education level, and occupation. The age at diagnosis, the type of bone tumour, the location of the tumour and relation to neurovascular structures as well as any metastases and comorbidities are registered.

In addition, the timing and type of surgery, along with the use of (neo)adjuvant chemotherapy, radiotherapy, recurrences, complications, and any further interventions, will be documented prospectively. Changes to the proposed surgical plan due to disease progression or preoperative complications will also be reported.

### Evaluation methods

#### Primary outcome

The study’s primary outcome is the level of perceived involvement in the decision-making process. For patients and parents, this will be assessed using the Shared Decision Making Questionnaire (SDM-Q-9) [29, 30], which will be compared with the perceived level of involvement measured during the 2015–2016 baseline survey.

### Secondary outcome

The study’s secondary outcomes include preferred role in making decisions, decisional conflict, decisional regret, daily functioning, and quality of life. They will be assessed with the Control Preferences Scale (CPS) [31, 32], Decisional Conflict Scale (DCS) [33, 34], Decisional Regret Scale (DRS) [35], Toronto Extremity Salvage Score – Lower Extremity (TESS - LE) [36], Bone Tumour (Bt)-DUX [37], Utrecht Scale for Evaluation of Rehabilitation – Participation (USER-P) [38, 39], Paediatric Quality of Life Inventory Generic Core Scales (PedsQL GCS) [40], and the ‘Perceived physical appearance’ subscale of the Paediatric Quality of Life Inventory Cancer Module (PedsQL CM) [41]. The Body Image Scale (BIS) is used for individuals over 16 years of age [42]. The TNO-AZL Questionnaire for Adult’s Quality of Life (TAAQOL) is used for individuals aged 16 years or older [43]. The TNO-AZL Children’s Quality of Life Questionnaire (TACQOL) is used for patients between 6 and 15 years of age or for proxy reports [44, 45]. The TNO-AZL Preschool Children’s Quality of Life Questionnaire (TAPQOL) is used for proxy reports of children aged 0–5 years [46]. The questionnaires are administered at different time points, as shown in Table 1.

The SDM-Q-doc questionnaire and CPS are used to assess SDM from the orthopaedic surgeon’s perspective [29]. The questionnaires are completed after the fourth consultation, which is when the final surgical decision is made.

All four individual consultations by the orthopaedic surgeon will be evaluated by a modified version of the Observing Patient Involvement (OPTION-5) instrument developed by Elwyn et al. [47, 48]. This five-item observer-based tool measures the level of patient involvement in clinical decision-making. A nurse practitioner or independent researcher will observe and complete the checklist after each consultation. The nurse practitioner will be trained in observing and completing the checklist. The final score will be based on the highest score for each item.

SDM related questionnaires, such as the SDM-Q-9, CPS, and DCS, were slightly adapted to be suitable for the specific surgical situation.

**Table 1:** Overview of questionnaires used to assess SDM for patients and parents, at different time points

Questionnaires	T1	T2	T3	Applicability patients (age)	Applicability parents
SDM-Q-9	X	X	X	10–26 years	parents
CPS	X	X	X	10–26 years	parents
DCS	X	X	X	10–26 years	parents
DRS		X	X	10–26 years	parents
TESS		X	X	10–26 years	parents
Bt-DUX		X	X	10–26 years	parents
USER-P		X	X	10–26 years	parents
PedsQL GCS		X	X	10–26 years	parents
Subscale PedsQL CM		X	X	10–26 years	-
BIS		X	X	16–26 years	-
TAAQOL			X	16–26 years	-
TACQOL			X	6–16 years	parents
TAPQOL			X	-	parents

T1 = before surgery (between consultation 4 and surgery); T2 = 1 year after surgery; T3 = 2 years after surgery; SDM-Q-9 = Shared Decision Making Questionnaire; CPS = Control Preferences Scale; DCS = Decisional Conflict Scale; DRS = Decisional Regret Scale; TESS - LE = Toronto Extremity Salvage Score - Lower Extremity; Bt-DUX = Bone Tumour-DUX; USER-P = Utrecht Scale for Evaluation of Rehabilitation - Participation; PedsQL GCS = Paediatric Quality of Life Inventory Generic Core Scales; PedsQL CM = Paediatric Quality of Life Inventory Cancer Module; BIS = Body Image Scale; TAAQOL = TNO-AZL Questionnaire for Adult’s Quality of Life; TACQOL = TNO-AZL Children’s Quality of Life Questionnaire; TAPQOL = TNO-AZL Preschool Children’s Quality of Life Questionnaire

### Optimizing the SDM model

#### Satisfaction questionnaire

Patient satisfaction with the decision-making process and decision aid will be evaluated. In addition, patients and their parents will be queried regarding any missing information or suggestions for enhancing the model. The satisfaction of patients and parents with the decision-making process will be assessed through a single question (Appendix 3). The adequacy of the information provided regarding surgical decisions will be evaluated using a series of questions (Appendix 4–5).

#### Interviews

Semi-structured in-depth interviews will be conducted with patients and parents to identify potential areas for improving the implemented model. The interview topics will focus on satisfaction with the decision-making process, consultations with the orthopaedic surgeon, physical therapist, and rehabilitation physician, decision-making, information on surgical options, and involvement of the child (for parental queries). The interview guide is presented in Appendix 6. The interviews will be conducted approximately one year after surgery, with each session lasting approximately 30 min. Sessions will be audio-recorded and transcribed verbatim. The study will continue until saturation is reached, with a target of 10 patients and 10 parents, facilitating a comprehensive understanding of the decision-making process and the conditions necessary for optimization.

The implemented model will also undergo evaluation by healthcare professionals involved in the care process. A questionnaire will be administered to the orthopaedic surgeon, (paediatric) oncologist, nurse practitioner, rehabilitation physician, physical therapist, and psychologist, who have been engaged with one or more patients at the end of the study (Appendix 7).

### Clinical data collection

The demographics and the questionnaires data are collected in Castor EDC, a web-based electronic data capture platform for clinical research [49]. At inclusion, patients are registered in Castor via a record ID. A separate subject identification list is kept password-protected by the study coordinator and principal investigator. The data collected include patient characteristics such as age and sex, oncological diagnosis, and treatment. Patient-Reported Outcome Measures (PROMs) are presented to patients and parents via the KLIK (Quality of Life in Clinical Practice) PROM portal, a web-based platform designed for completing PROM assessments or on paper, depending on the centre [50]. To facilitate secure delivery of pseudonymised data from the KLIK PROM portal at the closure of the study, the subject’s record ID is entered into his/her account by the study coordinators upon enrolment.

The interviews will be voice recorded and transcribed verbatim. The voice records of the interviews will be deleted immediately after transcription. The transcribed interviews will be de-identified. The transcriptions will be coupled to the subject’s records ID stored on the local G drive of the hospital.

### Privacy

Access to the Castor database is secured by log-in credentials and is restricted to the principal investigator (JAMB), and study coordinator (KJB). The data only contain pseudonymised data and are entered into the database by the study coordinator. As per hospital guidelines, data will be stored for 15 years after study closure. At the closure of the study, a pseudonymised export file including all the data will be generated by the KLIK PROM team based on the record ID’s and informed consent forms of the subjects. The completed paper study forms will be stored in a locked cabinet.

### Data analysis

Descriptive statistics will be provided for the entire group and relevant subgroups. Categorical variables will be presented as frequencies (n) and percentages (%). Continuous variables will be plotted graphically to assess the distribution and determine the most appropriate method for data presentation and analysis. The mean and standard deviation will be reported if the variables are normally distributed. Medians and interquartile ranges (IQRs) will be reported in the case of violations of normality.

The role of patients, parents, and other stakeholders and their appreciation of this role during the SDM process in malignant bone tumour surgery will be presented mainly descriptively in terms of frequencies, means and standard deviations. The results of the questionnaire on the level of involvement, decisional stress and decisional regret will be compared with the baseline measurements conducted among patients, parents, and stakeholders from the four national orthopaedic oncology centres before the implementation of SDM in 2015–2016 using the same measurement instruments. The unpaired t-test will be used to compare the two groups. As multiple testing comparisons will be conducted, the significance threshold will be adjusted using the Bonferroni correction. In addition to comparing the groups, the Pearson’s correlation coefficient between the mean scores of the SDM-Q-9, the Decisional Conflict Scale (DCS), and the Decisional Regret Scale (DRS) will be estimated. In the case of deviation from the normal distribution, Spearman’s correlation coefficient will be used.

Due to the presence of repeated measurements among the same participants, we will employ a mixed model to study the effect of time on three outcomes: the SDM-Q-9, DCS and DRS. Additionally, we will illustrate these trajectories using graphs depicting the mean scores for each outcome at each time point along with 95% confidence intervals.

Linear regression models will be used to determine whether there is an association between the three outcome measures (perceived involvement, decisional stress, and decisional regret) and predictors such as age, sex, educational level, type of surgery, ethnicity, and complications.

Pearson’s or Spearman’s correlation coefficients will be used for the SDM-Q-9, DCS and DRS, as well as for studying the relationships between patient, parents, and their orthopaedic surgeon scores.

The mean scores for quality of life and daily functioning will be compared with the Dutch reference score. In addition, they will be compared with the results of previous studies conducted in this population. These comparisons will be performed using two-sample t-tests to assess any significant differences between our sample data and the reference scores or previous study results.

Statistical significance was set at *p* < 0.05 (SPSS version 26.0). Statistical analyses will be performed using SPSS (IBM Corp. Released 2019. IBM SPSS Statistics for Windows, Version 26.0. Armonk, NY: IBM Corp).

The transcripts of the interviews will be analysed in the software program, MAXQDA. Several MAXQDA functions will be used to support the different steps of the analysis process and to ensure that the analyses are both systematic and transparent. These functions include coding, classification, and memos. The qualitative analysis will follow the inductive thematic analysis approach [54]. The following stages will be completed: familiarizing, generating codes, combining codes to themes, reviewing themes, determining theme significance, and reporting findings (Appendix 8). The final step is to finalize and structure the themes to describe and present the findings. This is a dynamic process in which it is possible to return to earlier stages.

### Ethical considerations

#### Research ethics

The Medical Research Ethics Committee of the Amsterdam University Medical Centre (Amsterdam, the Netherlands) confirmed that the Medical Research Involving Human Subjects Act does not apply to this study.

### Consent

According to Dutch ethical regulations, informed consent must be obtained from both parents or guardians for patients aged 15 years and younger. For patients from the age of 12, informed consent from the patient is also required. Patients aged 16 years and older are entitled to give their own consent. Parents also gave their informed consent for their own participation.

### Dissemination

After the study is closed, the list of subject identifications will be kept at the local centres. The database will be stored in the orthopaedic department of the Amsterdam University Medical Centre. Any future requests for the use of study data will be assessed by the principal investigator (JAMB). The study coordinator (KJB) will report the results of the study as soon as possible after finalization of the data analysis through publications. We will adhere to the guidelines of the publishing journal for determination of the authorships.

## Discussion

We aim to make a meaningful contribution to the literature on SDM in orthopaedic oncology for children and young adults. The task of selecting the optimal surgical approach for patients, especially those with primary malignant bone tumours around the knee, poses a considerable challenge. These patients face life-altering surgery and must navigate through various surgical options, each carrying distinct implications for rehabilitation, functionality, and cosmetic outcomes. Given the gravity of these decisions and the preferred involvement of patients with a primary malignant bone tumour and their parents, involving them in the surgical decision-making process is paramount [27].

Advances in chemotherapy regimens have significantly improved survival rates and shifted the focus towards optimizing surgical outcomes. In the late 1970s, around 80% of patients underwent amputation, but now, thanks to neoadjuvant chemotherapy, improved imaging, and progression in surgical techniques, limb-saving surgery can be performed in 85% of patients. Although limb-saving surgery seems like the best solution at first glance, each surgical option represents a life-altering surgical intervention, with varying implications for complication risks, rehabilitation, and functionality. All options are associated with specific risks and complications. Infection, mechanical failure, and the need for revision surgery are not uncommon after limb-saving surgery. Studies have shown that the survival of knee tumour mega prostheses varies, with failure rates depending on factors such as implant design, patient activity level, and adjuvant treatments [51, 52]. Grimer et al. showed that around 16% of patients who undergo limb-saving surgery may eventually require amputation [53]. Beyond complications, the rehabilitation process also varies between the surgical options. Biological reconstructions often require long periods of no weight-bearing, while endoprosthesis reconstruction allows immediate weight-bearing. However, in the long term, biological reconstructions generally have no restrictions on functionality, whereas with an endoprosthesis, activities like contact sports may no longer be possible. Weighing the future functional abilities and limitations, rehabilitation trajectory, and potential complications of the different surgical options is crucial for shared decision-making.

To address this need, we developed and implemented an SDM model alongside a supportive decision aid for young bone sarcoma patients as part of our standard care protocol. Our implementation involves analysing the impact of this model on the surgical decision-making process, with a commitment to continuously optimizing it as part of our standard care approach.

This study has limitations, mainly due to its focus on patients with primary malignant bone tumours around the knee, which impacts the study’s sample size. However, by sharing our study protocol with the international community, we aim to expand the data set by initiating collaborative studies to enrich the data and further optimize the SDM model. As has been developed for our specific Dutch setting, the SDM model may present challenges when applied in different contexts. However, it provides a foundational framework that can be adapted to various settings.

Our goal is to empower patients to make informed decisions and foster realistic expectations regarding their surgical outcomes. By promoting the active involvement of patients and their parents in the decision-making process, we seek to optimize the selection of the most suitable surgical options, reduce decisional regret, and ultimately improve patients’ long-term quality of life. Sharing our implementation and study protocol may serve as a blueprint for other centres looking to adopt SDM methods in an era where patients seek autonomy and reliable information.

By sharing our protocol, we aim to conduct future collaborative studies to expand the dataset and explore its implementation in various healthcare settings and cultural contexts. Future developments could then focus on expanding the SDM model to other bone tumour locations. By continuing to refine and disseminate our approach, we hope to contribute to a more patient-centred paradigm in orthopaedic oncology, ultimately enhancing the quality of care provided to this vulnerable population.

## Electronic supplementary material

Below is the link to the electronic supplementary material.

Supplementary Material 1

Supplementary Material 2

Supplementary Material 3

Supplementary Material 4

Supplementary Material 5

Supplementary Material 6

Supplementary Material 7

Supplementary Material 8

## Data Availability

No datasets were generated or analysed during the current study.

## Abbreviations

QoL: Quality of life
SDM: Shared decision making
MRI: Magnetic resonance imaging
SDM-Q-9: Shared decision Making Questionnaire
SDM-Q-Doc: Physician version of the SDM-Q-9
CPS: Control Preferences Scale
OPTION-5: Observing Patient Involvement
DCS: Decisional Conflict Scale
DRS: Decisional Regret Scale
TESS: Toronto Extremity Salvage Score
Bt-DUX: Bone tumour-DUX
USER-P: Utrecht Scale for Evaluation of Rehabilitation – Participation
PedsQL GCS: Paediatric Quality of Life Inventory Generic Core Scales
PedsQL CM: Paediatric Quality of Life Inventory Cancer Module
BIS: Body Image Scale
TAAQOL: TNO-AZL Questionnaire for Adult’s Quality of Life
TACQOL: TNO-AZL Children’s Quality of Life Questionnaire
TAPQOL: TNO-AZL Preschool Children’s Quality of Life Questionnaire
EDC: Electronic Data Capture
KLIK: Quality of Life in Clinical Practice
PROM: Patient-Reported Outcome Measure
IQR: Interquartile range

## References

[1] Stiller CA, et al. Bone tumours in European children and adolescents, 1978–1997. Report from the Automated Childhood Cancer Information System project. Eur J Cancer. 2006;42(13):2124–35. 16919776 10.1016/j.ejca.2006.05.015

[2] Damron TA, Ward WG, Stewart A. Osteosarcoma, chondrosarcoma, and Ewing’s sarcoma: National Cancer Data Base Report. Clin Orthop Relat Res. 2007;459:40–7. 17414166 10.1097/BLO.0b013e318059b8c9

[3] Bielack S, Carrle D, Casali PG. Osteosarcoma: ESMO clinical recommendations for diagnosis, treatment and follow-up. Ann Oncol. 2009;20(Suppl 4):137–9. 19454435 10.1093/annonc/mdp154

[4] Paulussen M, et al. Ewing’s sarcoma of the bone: ESMO clinical recommendations for diagnosis, treatment and follow-up. Ann Oncol. 2009;20(Suppl 4):140–2. 19454436 10.1093/annonc/mdp155

[5] Abed R, Grimer R. Surgical modalities in the treatment of bone sarcoma in children. Cancer Treat Rev. 2010;36(4):342–7. 20223595 10.1016/j.ctrv.2010.02.010

[6] Veth R, et al. Limb salvage in musculoskeletal oncology. Lancet Oncol. 2003;4(6):343–50. 12788406 10.1016/s1470-2045(03)01114-8

[7] Wafa H, Grimer RJ. Surgical options and outcomes in bone sarcoma. Expert Rev Anticancer Ther. 2006;6(2):239–48. 16445376 10.1586/14737140.6.2.239

[8] Wilkins RM, Camozzi AB, Gitelis SB. Reconstruction options for pediatric bone tumors about the knee. J Knee Surg. 2005;18(4):305–9. 16262014 10.1055/s-0030-1248197

[9] Nagarajan R, et al. Limb salvage and amputation in survivors of pediatric lower-extremity bone tumors: what are the long-term implications? J Clin Oncol. 2002;20(22):4493–501. 12431974 10.1200/JCO.2002.09.006

[10] Bekkering WP et al. Quality of life after bone sarcoma surgery around the knee: a long-term follow-up study. Eur J Cancer Care (Engl), 2017. 26(4). 10.1111/ecc.12603 28657211

[11] Bekkering WP, et al. Quality of life, functional ability and physical activity after different surgical interventions for bone cancer of the leg: a systematic review. Surg Oncol. 2012;21(2):e39–47. 21974808 10.1016/j.suronc.2011.09.002

[12] Akahane T, et al. Evaluation of postoperative general quality of life for patients with osteosarcoma around the knee joint. J Pediatr Orthop B. 2007;16(4):269–72. 17527105 10.1097/BPB.0b013e3280925670

[13] Hopyan S, et al. Function and upright time following limb salvage, amputation, and rotationplasty for pediatric sarcoma of bone. J Pediatr Orthop. 2006;26(3):405–8. 16670557 10.1097/01.bpo.0000203016.96647.43

[14] Aksnes LH, et al. Limb-sparing surgery preserves more function than amputation: a scandinavian sarcoma group study of 118 patients. J Bone Joint Surg Br. 2008;90(6):786–94. 18539673 10.1302/0301-620X.90B6.19805

[15] Zahlten-Hinguranage A, et al. Equal quality of life after limb-sparing or ablative surgery for lower extremity sarcomas. Br J Cancer. 2004;91(6):1012–4. 15292924 10.1038/sj.bjc.6602104 PMC2747710

[16] Reulen RC, et al. Health-status of adult survivors of childhood cancer: a large-scale population-based study from the British Childhood Cancer Survivor Study. Int J Cancer. 2007;121(3):633–40. 17405119 10.1002/ijc.22658

[17] Koopman HM, et al. Health-related quality of life and coping strategies of children after treatment of a malignant bone tumor: a 5-year follow-up study. Pediatr Blood Cancer. 2005;45(5):694–9. 15924359 10.1002/pbc.20408

[18] Bekkering WP, et al. Quality of life in young patients after bone tumor surgery around the knee joint and comparison with healthy controls. Pediatr Blood Cancer. 2010;54(5):738–45. 20127850 10.1002/pbc.22439

[19] Stiggelbout AM, et al. Shared decision making: really putting patients at the centre of healthcare. BMJ. 2012;344:e256. 22286508 10.1136/bmj.e256

[20] Kaafarani HM. Surgeon preference and variation of surgical care. Am J Surg. 2011;201(5):709–11. 21356529 10.1016/j.amjsurg.2010.03.006

[21] WGBO.

[22] Salzburg statement on shared decision making. BMJ, 2011. 342: p. d1745. 10.1136/bmj.d1745 21427038

[23] Stiggelbout AM, Pieterse AH, De Haes JC. Shared decision making: concepts, evidence, and practice. Patient Educ Couns. 2015;98(10):1172–9. 26215573 10.1016/j.pec.2015.06.022

[24] Elwyn G, et al. A three-talk model for shared decision making: multistage consultation process. BMJ. 2017;359:j4891. 29109079 10.1136/bmj.j4891 PMC5683042

[25] Joosten EA, et al. Systematic review of the effects of shared decision-making on patient satisfaction, treatment adherence and health status. Psychother Psychosom. 2008;77(4):219–26. 18418028 10.1159/000126073

[26] van Stam MA, et al. Shared decision making in prostate Cancer Care-encouraging every patient to be actively involved in decision making or ensuring the patient Preferred Level of involvement? J Urol. 2018;200(3):582–9. 29501555 10.1016/j.juro.2018.02.3091

[27] Blom K, et al. Shared decision making in primary malignant bone tumour surgery in children and young adults. EJC Pediatr Oncol. 2024;3:100138. 10.1186/s13018-024-05192-y PMC11531153 39487545

[28] van de Sande MAJ, et al. *Van Nes-Borggreve Rotationplasty okneee Knee*, in *European Surgical Orthopaedics and Traumatothey: The EFORT Textbook*. Berlin Heidelberg: Berlin, Heidelberg: Springer; 2014. pp. 4135–47. G. Bentley, Editor.

[29] Rodenburg-Vandenbussche S, et al. Dutch translation and psychometric testing of the 9-Item Shared decision making questionnaire (SDM-Q-9) and Shared decision making questionnaire-physician version (SDM-Q-Doc) in primary and secondary care. PLoS ONE. 2015;10(7):e0132158. 26151946 10.1371/journal.pone.0132158 PMC4494856

[30] Kriston L, et al. The 9-item Shared decision making questionnaire (SDM-Q-9). Development and psychometric properties in a primary care sample. Patient Educ Couns. 2010;80(1):94–9. 19879711 10.1016/j.pec.2009.09.034

[31] Kremer H, Ironson G. Measuring the involvement of people with HIV in treatment decision making using the Control preferences Scale. Med Decis Making. 2008;28(6):899–908. 18757843 10.1177/0272989X08317014

[32] Degner LF, Sloan JA, Venkatesh P. The Control preferences Scale. Can J Nurs Res. 1997;29(3):21–43. 9505581

[33] Koedoot N, et al. The decisional conflict scale: further validation in two samples of Dutch oncology patients. Patient Educ Couns. 2001;45(3):187–93. 11722854 10.1016/s0738-3991(01)00120-3

[34] O’Connor AM. Validation of a decisional conflict scale. Med Decis Mak. 1995;15(1):25–30. 10.1177/0272989X9501500105 7898294

[35] Brehaut JC, et al. Validation of a decision regret scale. Med Decis Mak. 2003;23(4):281–92. 10.1177/0272989X03256005 12926578

[36] Davis AM, et al. Development of a measure of physical function for patients with bone and soft tissue sarcoma. Qual Life Res. 1996;5(5):508–16. 8973131 10.1007/BF00540024

[37] Bekkering WP, et al. The Bt-DUX: development of a subjective measure of health-related quality of life in patients who underwent surgery for lower extremity malignant bone tumor. Pediatr Blood Cancer. 2009;53(3):348–55. 19459200 10.1002/pbc.22078

[38] van der Zee CH, et al. Responsiveness of four participation measures to changes during and after outpatient rehabilitation. J Rehabil Med. 2011;43(11):1003–9. 22031346 10.2340/16501977-0879

[39] Post MW, et al. Development and validation of the Utrecht Scale for Evaluation of Clinical Rehabilitation (USER). Clin Rehabil. 2009;23(10):909–17. 19717505 10.1177/0269215509341524

[40] Varni JW, Seid M, Kurtin PS. PedsQL 4.0: reliability and validity of the Pediatric Quality of Life Inventory version 4.0 generic core scales in healthy and patient populations. Med Care. 2001;39(8):800–12. 11468499 10.1097/00005650-200108000-00006

[41] Varni JW, et al. The PedsQL in pediatric cancer: reliability and validity of the Pediatric Quality of Life Inventory Generic Core Scales, multidimensional fatigue scale, and Cancer Module. Cancer. 2002;94(7):2090–106. 11932914 10.1002/cncr.10428

[42] Hopwood P, et al. A body image scale for use with cancer patients. Eur J Cancer. 2001;37(2):189–97. 11166145 10.1016/s0959-8049(00)00353-1

[43] Bruil J, Vogels FM. The validity and reliability of the TAAQOL: a health-related quality of life instruments comprising health status weighted by the impact of problems on well being. Qual Life Res. 2001;10(3):257.

[44] Verrips G, et al. Health-related quality of life measure for children-the TACQOL+. J Appl Ther. 1997;1:357–60.

[45] Vogels T, et al. Measuring health-related quality of life in children: the development of the TACQOL parent form. Qual Life Res. 1998;7(5):457–65. 9691725 10.1023/a:1008848218806

[46] Fekkes M, et al. Development and psychometric evaluation of the TAPQOL: a health-related quality of life instrument for 1-5-year-old children. Qual Life Res. 2000;9(8):961–72. 11284215 10.1023/a:1008981603178

[47] Elwyn G, et al. Using a ‘talk’ model of shared decision making to propose an observation-based measure: Observer OPTION 5 item. Patient Educ Couns. 2013;93(2):265–71. 24029581 10.1016/j.pec.2013.08.005

[48] Stubenrouch FE, et al. OPTION(5) versus OPTION(12) instruments to appreciate the extent to which healthcare providers involve patients in decision-making. Patient Educ Couns. 2016;99(6):1062–8. 26776490 10.1016/j.pec.2015.12.019

[49] Castor EDC. *Castor Electronic Data Capture; 2019 [cited 29 Sep 2023]. Available: 560* https://www.castoredc.com/

[50] Schepers SA, et al. Real-world implementation of electronic patient-reported outcomes in outpatient pediatric cancer care. Psychooncology. 2017;26(7):951–9. 27502744 10.1002/pon.4242

[51] Pala E, et al. Megaprosthesis of the knee in tumor and revision surgery. Acta Biomed. 2017;88(2s):129–38. 28657574 10.23750/abm.v88i2-S.6523 PMC6179001

[52] Pala E, et al. Survival of modern knee tumor megaprostheses: failures, functional results, and a comparative statistical analysis. Clin Orthop Relat Res. 2015;473(3):891–9. 24874116 10.1007/s11999-014-3699-2 PMC4317408

[53] Grimer RJ, et al. Very long-term outcomes after endoprosthetic replacement for malignant tumours of bone. Bone Joint J. 2016;98–b(6):p857–64. 10.1302/0301-620X.98B6.37417 27235533

[54] Brown V and Clark V. Using thematic analysis in psychology, Qualitative Research in Psychology, 2006: 3(2), 77-101

