# Supplementary material for: Shared decision making in primary malignant bone tumour surgery around the knee in children and young adults: protocol for a prospective study
Source: J Orthop Surg Res. 2024 Nov 2;19:714. doi: 10.1186/s13018-024-05192-y (PMC11531153; doi:10.1186/s13018-024-05192-y)
Supplement: Supplementary file 1 — Supplementary Material 1 [file 13018_2024_5192_MOESM1_ESM.docx]

**Appendix 2**

| **cycle** |  |  | **1** |  |  |  |  | **2** |  |  |  | S U R G E R Y |
| --- | --- | --- | --- | --- | --- | --- | --- | --- | --- | --- | --- | --- |
|  |  | | | | |  |  |  |  |  |  |  |
|  | **A P** |  |  | M | M | **A P** |  |  | M | M |  |  |
|  |  |  |  |  |  |  |  |  |  |  |  |  |
| **week** | **1** | 2 | 3 | 4 | 5 | **6** | 7 | 8 | 9 | 10 |  |  |
|  |  |  |  |  |  |  |  |  |  |  |  |  |
|  |  |  |  |  |  |  |  |  |  |  |  | 11 |

| Consultation 4  Consultation 3  Consultation 2  Consultation 1  Physical therapist & rehabilitation doctor  Treatment schedule Osteosarcoma (Euramos protocol) | | |  |
| --- | --- | --- | --- |
|  |  |  |  |

|  |  |  |  |  |  |  |  |  |  |  |  |  |  |  |  |  |  |  |  |  |  |  |
| --- | --- | --- | --- | --- | --- | --- | --- | --- | --- | --- | --- | --- | --- | --- | --- | --- | --- | --- | --- | --- | --- | --- |
|  |  | | | | |  |  |  |  |  |  |  |  |  |  |  |  |  |  |  | S  U  R  G  E  R  Y |  |
|  |  |  |  |  |  |  |  |  |  |  |  |  |  |  |  |  |  |  |  |  |  |  |
|  | V D C |  | IE |  | V D C |  | IE |  | V D C |  | IE |  | V D C |  | IE |  | V D C |  |  |  |  |  |
|  |  |  |  |  |  |  |  |  |  |  |  |  |  |  |  |  |  |  |  |  |  |  |
|  |  |  |  |  |  |  |  |  |  |  |  |  |  |  |  |  |  |  |  |  |  |  |
|  |  |  |  |  |  |  |  |  |  |  |  |  |  |  |  |  |  |  |  |  |  |  |
| **week** | 1 | 2 | 3 | 4 | 5 | 6 | 7 | 8 | 9 | 10 | 11 | 12 | 13 | 14 | 15 | 16 | 17 | 18 | 19 |  |  |  |
|  |  |  |  |  |  |  |  |  |  |  |  |  |  |  |  |  |  |  |  |  | 20 |  |
|  |  |  |  |  |  |  |  |  |  |  |  |  |  |  |  |  |  |  |  |  |  | |

Physical therapist & rehabilitation doctor

Consultation 1

Consultation 2

Consultation 3

Consultation 4

Treatment schedule Ewing Sarcoma (EuroEwing 2012 protocol)
