# Supplementary material for: Shared decision making in primary malignant bone tumour surgery around the knee in children and young adults: protocol for a prospective study
Source: J Orthop Surg Res. 2024 Nov 2;19:714. doi: 10.1186/s13018-024-05192-y (PMC11531153; doi:10.1186/s13018-024-05192-y)
Supplement: Supplementary file 2 — Supplementary Material 2 [file 13018_2024_5192_MOESM2_ESM.docx]

**Appendix 6**

*Satisfaction*

Main question: How did you feel about being involved in the surgical decision-making process?

*Consultations orthopaedic surgeon*

Main question: Can you tell me how you experienced the consultations with the orthopaedic surgeon?

*Consultation(s) rehabilitation physician, physical therapist, healthcare professionals and others*

Main question: How did you experience the consultations with the physical therapist and rehabilitation physician?

Main question: Did you have any conversations with other healthcare professionals about making the decision?

- If yes, with whom?
- How did you experience these conversations?

Main question: Were any other people important to you during the decision-making process?

- If yes, who, and why were these people important to you?

*Decision making*

Main question: Can you describe how, in your opinion, the surgical decision was made?

*Information surgical options*

Main question: What did you think of the information you received about the surgical options?

What did you think of the decision aid that was offered?

*Child involvement (question only for parents)*

Main question: How did you perceive your child's involvement in making the decision?
