# Supplementary material for: Shared decision making in primary malignant bone tumour surgery around the knee in children and young adults: protocol for a prospective study
Source: J Orthop Surg Res. 2024 Nov 2;19:714. doi: 10.1186/s13018-024-05192-y (PMC11531153; doi:10.1186/s13018-024-05192-y)
Supplement: Supplementary file 3 — Supplementary Material 3 [file 13018_2024_5192_MOESM3_ESM.docx]

**Appendix 3**

Question satisfaction:

Are you satisfied with the way the choice of surgery was made?

- Very satisfied
- Satisfied
- Not unsatisfied / not satisfied
- Unsatisfied
- Very unsatisfied
