# Supplementary material for: Shared decision making in primary malignant bone tumour surgery around the knee in children and young adults: protocol for a prospective study
Source: J Orthop Surg Res. 2024 Nov 2;19:714. doi: 10.1186/s13018-024-05192-y (PMC11531153; doi:10.1186/s13018-024-05192-y)
Supplement: Supplementary file 5 — Supplementary Material 5 [file 13018_2024_5192_MOESM5_ESM.docx]

**Appendix 7**

**Questionnaire orthopaedic surgeons**

|  | Strongly disagree | Disagree | Somewhat disagree | Somewhat agree | Agree | Strongly agree |
| --- | --- | --- | --- | --- | --- | --- |
| 1. I think it is important for patients/parents to be involved in the choice of surgery | 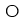 | 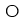 | 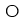 | 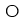 | 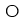 | 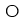 |
| 1. Patients over 18 years old can participate in thinking about/deciding on the surgery | 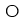 | 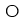 | 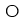 | 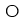 | 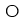 | 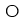 |
| 1. Children aged 12 to 18 can participate in thinking about/deciding on the surgery | 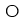 | 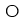 | 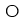 | 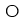 | 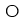 | 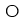 |
| 1. Children aged 8 to 12 can participate in thinking about/deciding on the surgery | 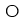 | 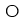 | 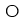 | 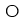 | 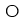 | 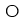 |
| 1. Children younger than 8 years can participate in thinking about/deciding on the surgery | 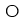 | 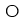 | 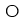 | 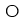 | 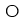 | 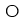 |
| 1. It is important for children aged 8 to 12 to be present when surgery is discussed | 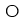 | 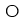 | 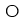 | 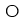 | 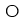 | 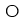 |
| 1. It is important for children under 8 years old to be present when surgery is discussed | 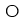 | 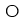 | 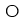 | 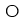 | 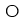 | 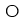 |
| 1. There is a risk of conflict (between parents and patient) when patients/parents are allowed to co-decide on the surgery | 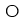 | 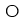 | 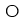 | 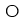 | 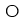 | 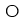 |

|  | Strongly disagree | Disagree | Somewhat disagree | Somewhat agree | Agree | Strongly agree |
| --- | --- | --- | --- | --- | --- | --- |
| 1. There is a risk of conflict (between patient/parents and orthopaedic surgeon) when patients/parents are allowed to co-decide on the surgery | 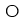 | 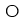 | 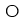 | 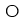 | 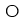 | 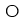 |
| 1. I find it difficult to involve the patient/parents in the decision about the surgery | 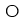 | 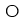 | 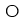 | 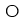 | 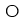 | 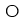 |
| 1. I think only the orthopaedic surgeon can make the decision on the surgery | 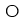 | 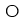 | 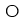 | 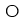 | 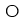 | 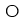 |
| 1. Before meeting with the patient/parents, I discuss surgical options in the multidisciplinary treatment team | 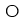 | 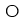 | 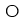 | 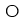 | 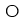 | 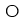 |
| 1. There is enough material for patient/parent education | 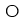 | 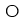 | 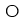 | 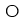 | 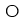 | 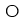 |
| 1. Shared Decision Making (SDM) contributes to making an informed choice | 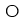 | 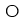 | 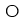 | 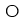 | 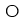 | 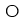 |
| 1. I find SDM useful when treating children/young adults with bone cancer | 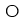 | 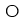 | 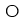 | 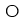 | 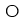 | 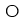 |

1. Which disciplines do you included in your decision-making?

(multiple answers are possible)

- (Paediatric) oncologist
- Radiologist
- Pathologist
- Rehabilitation doctor
- Physical therapist
- Nurse specialist
- Medical pedagogical workers
- Psychologist
- Plastic surgeon
- Instrument maker
- Other: …………………………………………..…………………………………………..

1. What other disciplines would you like to include?

(multiple answers are possible)

- (Paediatric) oncologist
- Radiologist
- Pathologist
- Rehabilitation doctor
- Physical therapist
- Nurse specialist
- Medical pedagogical workers
- Psychologist
- Plastic surgeon
- Instrument maker
- Other: …………………………………………..…………………………………………..

1. What materials do you use for preparation?

(multiple answers are possible)

- Photos
- Video / film
- Leaflets
- Contact with (former) patients
- Internet
- Self-made drawing
- Decision aid
- Conversation with physiotherapist/rehabilitation physician
- 3D model of the tumour
- Other: …………………………………………..…………………………………………..

1. What materials do you miss in preparation?

(multiple answers are possible)

- Photos
- Video / film
- Leaflets
- Contact with (former) patients
- Internet
- Self-made drawing
- Decision aid
- Conversation with physiotherapist/rehabilitation physician
- 3D model of the tumour
- Other: …………………………………………..…………………………………………..

1. Has the structured implementation of SDM changed the way you work?

- Yes
- No

1. Has applying SDM in a structured way given you a different perspective on SDM?

- Yes
- No

1. Are you now using the structured application of SDM for bone tumours other than tumours around the knee?

- Yes
- No

1. Do you feel that you involve the patient/parents more in the decision making than you did before the implementation of SDM?

- Yes
- No

1. Do you feel that the patient is satisfied with the decision-making process?

- Yes
- No 🡪 why not?

………………………………………………………………………………………………………………………………………………………………………………………………………………………………………………………………………………………………………………………………………………………………………………………………………………………

1. Do you feel that the parents are satisfied with the decision-making process?

- Yes
- No 🡪 why not?

………………………………………………………………………………………………………………………………………………………………………………………………………………………………………………………………………………………………………………………………………………………………………………………………………………………

1. Do you have any suggestions for improving the current decision-making process?

- No
- Yes, I would: ………………………………………………………………………………………………………………………………………………………………………………………………………………………………………………………………………………………………………………………………………………………………………………………………………………………

………………………………………………………………………………………………………………………………………………………………………………………………………………………………………………………………………………

**Questionnaire physical therapist and rehabilitation physician**

|  | Strongly disagree | Disagree | Somewhat disagree | Somewhat agree | Agree | Strongly agree |
| --- | --- | --- | --- | --- | --- | --- |
| 1. I think it is important for patients/parents to be involved in the choice of surgery | 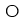 | 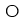 | 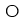 | 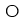 | 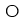 | 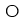 |
| 1. Patients over 18 years old can participate in thinking about/deciding on the surgery | 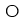 | 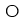 | 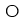 | 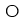 |  |  |
| 1. Children aged 12 to 18 can participate in thinking about/deciding on the surgery |  |  |  |  |  |  |
| 1. Children aged 8 to 12 can participate in thinking about/deciding on the surgery |  |  |  |  |  |  |
| 1. Children younger than 8 years can participate in thinking about/deciding on the surgery |  |  |  |  |  |  |
| 1. It is important for children aged 8 to 12 to be present when surgery is discussed |  |  |  |  |  |  |
| 1. It is important for children under 8 years old to be present when surgery is discussed |  |  |  |  |  |  |
| 1. There is a risk of conflict (between parents and patient) when patients/parents are allowed to co-decide on the surgery |  |  |  |  |  |  |
| 1. There is a risk of conflict (between patient/parents and orthopaedic surgeon) when patients/parents are allowed to co-decide on the surgery |  |  |  |  |  |  |

|  | Strongly disagree | Disagree | Somewhat disagree | Somewhat agree | Agree | Strongly agree |
| --- | --- | --- | --- | --- | --- | --- |
| 1. I think only the orthopaedic surgeon can make the decision on the surgery |  |  |  |  |  |  |
| 1. There is enough material for patient/parent education |  |  |  |  |  |  |
| 1. Shared Decision Making (SDM) contributes to making an informed choice |  |  |  |  |  |  |
| 1. I find SDM useful when treating children/young adults with bone cancer |  |  |  |  |  |  |

1. What materials do you use for preparation?

(multiple answers are possible)

- Photos
- Video / film
- Leaflets
- Contact with (former) patients
- Internet
- Self-made drawing
- Decision aid
- Conversation with physiotherapist/rehabilitation physician
- 3D model of the tumour
- Other: …………………………………………..

1. What materials do you miss in preparation?

(multiple answers are possible)

- Photos
- Video / film
- Leaflets
- Contact with (former) patients
- Internet
- Self-made drawing
- Decision aid
- Conversation with physiotherapist/rehabilitation physician
- 3D model of the tumour
- Other: …………………………………………..

1. Do you feel that the decision-making process has improved now that shared decision making in a structured way has been implemented?

- Yes
- No
- I can not judge

1. I would like to be present during discussions with the orthopaedic surgeon and the patient/family

- No
- Yes

If yes, why would you want to attend

(multiple answers are possible)

- - Support the patient and parents
  - To participate in the conversation
  - To know what is being told
  - Other, namely………………………………………………………………………………………………… …………………………………………………………………………………………………………………………………………………………………………………………………………………………………………………………

1. Do you feel that the patient is satisfied with the decision-making process?

- Yes
- No 🡪 why not?

………………………………………………………………………………………………………………………………………………………………………………………………………………………………………………………………………………

………………………………………………………………………………………………………………………………………

1. Do you feel that the parents are satisfied with the decision-making process?

- Yes
- No 🡪 why not?

………………………………………………………………………………………………………………………………………………………………………………………………………………………………………………………………………………………………………………………………………………………………………………………………………………………

1. Do you have any suggestions for improving the current decision-making process?

- No
- Yes, I would: ………………………………………………………………………………………………………………………………………………………………………………………………………………………………………………………………………………………………………………………………………………………………………………………………………………………

**Questionnaire health care professionals**

|  | Strongly disagree | Disagree | Somewhat disagree | Somewhat agree | Agree | Strongly agree |
| --- | --- | --- | --- | --- | --- | --- |
| 1. I think it is important for patients/parents to be involved in the choice of surgery |  |  |  |  |  |  |
| 1. Patients over 18 years old can participate in thinking about/deciding on the surgery |  |  |  |  |  |  |
| 1. Children aged 12 to 18 can participate in thinking about/deciding on the surgery |  |  |  |  |  |  |
| 1. Children aged 8 to 12 can participate thinking about/deciding on the surgery |  |  |  |  |  |  |
| 1. Children younger than 8 years can participate in thinking about/deciding on the surgery |  |  |  |  |  |  |
| 1. It is important for children aged 8 to 12 to be present when surgery is discussed |  |  |  |  |  |  |
| 1. It is important for children under 8 years old to be present when surgery is discussed |  |  |  |  |  |  |
| 1. There is a risk of conflict (between parents and patient) when patients/parents are allowed to co-decide on the surgery |  |  |  |  |  |  |
| 1. There is a risk of conflict (between patient/parents and orthopaedic surgeon) when patients/parents are allowed to co-decide on the surgery |  |  |  |  |  |  |

|  | Strongly disagree | Disagree | Somewhat disagree | Somewhat agree | Agree | Strongly agree |
| --- | --- | --- | --- | --- | --- | --- |
| 1. I think only the orthopaedic surgeon can make the decision on the surgery |  |  |  |  |  |  |
| 1. Shared Decision Making (SDM) contributes to making an informed choice |  |  |  |  |  |  |
| 1. I find SDM useful when treating children/young adults with bone cancer |  |  |  |  |  |  |

1. Do you feel involved in the decision-making process?

- Yes
- No (go to question 15)

1. If so, what do you feel involved in?

(multiple answers are possible)

- Providing information
- Listening
- Contact point – arranging things
- Other: ………………………………………………………………………………………………………………………

1. Would you like to be more involved?

- Yes
- No (go to question 17)

1. If so, what role would you like to have?

(multiple answers are possible)

- Providing information
- Listening
- Contact point – arranging things
- To know what is being told
- Patient advocate
- Other: …………………………………………………………………………………………………………………

1. What is the added value of or for your speciality in this decision-making process?

……………………………………………………………………………………………………………………………………………………………………………………………………………………………………………………………………………………………………………………………………………………………………………………………………………………………………………………………………………………………………………………………………………………………………………………………………………………………………………………………………………………………………………………………………………………………………………………………………………………………………………….

1. Do you feel that the decision-making process has improved now that shared decision making in a structured way has been implemented?

- Yes
- No
- I can not judge

1. Is there enough information?

- Yes
- No 🡪 what is missing?

………………………………………………………………………………………………………………………………………………………………………………………………………………………………………………………………………………………………………………………………………………………………………………………………………………………

1. I would like to be present at meetings with the orthopaedic surgeon and family.

- No
- Yes

If yes, why would you want to attend?

(multiple answers are possible)

- - Support the patient and parents
  - To participate in the conversation
  - To know what is being told
  - Other, namely.………………………………………………………………………………………………… …………………………………………………………………………………………………………………………………………………………………………………………………………………………………………………………

1. Do you feel that the patient is satisfied with the decision-making process?

- Yes
- No 🡪 why not?

………………………………………………………………………………………………………………………………………………………………………………………………………………………………………………………………………………

………………………………………………………………………………………………………………………………………

1. Do you feel that the parents are satisfied with the decision-making process?

- Yes
- No 🡪 why not?

………………………………………………………………………………………………………………………………………………………………………………………………………………………………………………………………………………

………………………………………………………………………………………………………………………………………

1. Do you have any suggestions for improving the current decision-making process?

- No
- Yes, I would: ………………………………………………………………………………………………………………………………………………………………………………………………………………………………………………………………………………………………………………………………………………………………………………………………………………………
