# Supplementary material for: Shared decision making in primary malignant bone tumour surgery around the knee in children and young adults: protocol for a prospective study
Source: J Orthop Surg Res. 2024 Nov 2;19:714. doi: 10.1186/s13018-024-05192-y (PMC11531153; doi:10.1186/s13018-024-05192-y)
Supplement: Supplementary file 6 — Supplementary Material 6 [file 13018_2024_5192_MOESM6_ESM.docx]

**Appendix 4**

**Questions general decision process**

1. Who first informed you about the surgery?

- Orthopaedic surgeon
- Oncologist
- Other: …………………………………………..…………………………………………..

1. By whom was this information later repeated / supplemented? (more than one answer is possible)

- Orthopaedic surgeon
- Oncologist
- Rehabilitation physician
- Medical pedagogical workers
- Physical therapist
- Nobody
- Other: …………………………………………..…………………………………………..

1. What options were mentioned by the doctor as possibilities?

(more than one answer is possible)

- Amputation
- Rotationplasty
- Internal prosthesis
- Growth prosthesis
- Donor bone
- Other: …………………………………………..…………………………………………..

1. Which of these was your preferred option?

(more than one answer is possible)

- Amputation
- Rotationplasty
- Internal prosthesis
- Growth prosthesis
- Donor bone
- No preference
- Other: …………………………………………..…………………………………………..

1. Who was of importance to you in making the decision?

(more than one answer is possible)

- Orthopaedic surgeon
- Oncologist
- Nurse specialist
- Physical therapist
- Rehabilitation physician
- Psychologist
- Social worker
- Medical pedagogical workers
- Physical therapist
- Other, namely: …………………………………………..…………………………………………..

|  | Strongly disagree | Disagree | | Somewhat disagree | | Somewhat agree | | Agree | | Strongly agree |  |
| --- | --- | --- | --- | --- | --- | --- | --- | --- | --- | --- | --- |
| 1. When making the choice of surgery, your opinion was considered to be as important as the doctor's opinion | 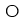 | 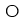 | | 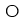 | | 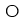 | | 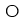 | | 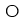 |  |
| 1. There was enough time for questions and dialogue | 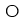 | 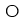 | | 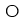 | | 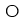 | | 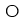 | | 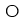 |  |
|  | Strongly disagree | | Disagree | | Neither agree nor disagree | | Agree | | Strongly agree | | |
| 1. I felt that I could co-decide on the surgery | 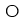 | | 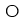 | | 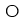 | | 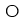 | | 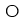 | | |
| 1. I liked being involved in the choice of surgery | 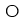 | | 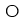 | | 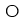 | | 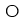 | | 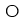 | | |
| 1. I knew which was the best choice for me | 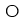 | | 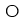 | | 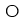 | | 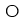 | | 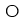 | | |

**Questions information surgical options**

1. How was this information given? (more than one answer is possible)

- Oral
- Photographs
- Movie
- Decision aid
- Online
- (former) patients
- Other: …………………………………………..………………………………………..………………………………………

1. In what supplementary way would you have liked to receive the information?

(more than one answer is possible)

- Oral
- Photographs
- Movie
- Online
- (former) patients
- No other way
- Other:…………………………………………..………………………………………..………………………………………

1. What information was given about the surgery?

(more than one answer is possible)

- The surgery itself (technical information)
- The situation after the surgery
- The rehabilitation/post-treatment in hospital and after discharge
- The impact on daily life (such as walking, school, work)
- The implications for sports
- The cosmetic consequences (scar, missing leg)
- The leg prosthesis (after amputation or rotationplasty)
- The risk for the future after sparing surgery (for example: the risk of fracture after donor bone surgery or loosening of an internal prosthesis)
- Relationships and sexuality
- Other: …………………………………………..………………………………………..………………………………………

1. Looking back, what information did you miss? Information on:

(more than one answer is possible)

- The surgery itself (technical information)
- The situation after the surgery
- The rehabilitation/post-treatment in hospital and after discharge
- The impact on daily life (such as walking, school, work)
- The implications for sports
- The cosmetic consequences (scar, missing leg)
- The leg prosthesis (after amputation or rotationplasty)
- The risk for the future after sparing surgery (for example: the risk of fracture after donor bone surgery or loosening of a joint prosthesis)
- Relationships and sexuality
- Didn’t miss anything
- Other: …………………………………………..………………………………………..………………………………………

1. Were you also told about the negative consequences/risks of the surgery?

- Yes
- No

1. Do you think you should have been told more of these negative consequences/risks before surgery?

- No
- Yes, namely: …………………………………………..………………………………………………………………………

……………………………………………………………………………………………………………………………………….

……………………………………………………………………………………………………………………………………….

1. What information was decisive in making your choice? (please choose one answer)

- The impact on daily life (such as walking, school, work)
- The impact on sports
- The cosmetic consequences (scar, missing leg)
- The vulnerability of the leg after sparing surgery (breaking, loosening)
- Being dependent on a leg prosthesis (in an amputation or rotationplasty)
- The necessary surgeries in the future after sparing surgery (wear and tear)
- Other: …………………………………………..………………………………………..………………………………………

**Questions decision aid**

1. Did you look at the decision aid?

- Yes, alone
- Yes, with my parent(s)
- No (go to question 22)

1. Was the information in the decision aid clear?

- Yes
- No, namely: …………………………………………..………………………………………………………………………

……………………………………………………………………………………………………………………………………….

……………………………………………………………………………………………………………………………………….

1. Did you feel the decision aid added anything to the information you received from the orthopaedic surgeon?

- Yes
- No

1. Did the decision aid help you to make the decision?

- Yes
- No

1. Is there anything else you would like to tell us after completing the questionnaire?

- No
- Yes, namely: …………………………………………..………………………………………………………………………

……………………………………………………………………………………………………………………………………….

……………………………………………………………………………………………………………………………………….
