# Supplementary figures and images for: Shared decision making in primary malignant bone tumour surgery around the knee in children and young adults: protocol for a prospective study
Source: J Orthop Surg Res. 2024 Nov 2;19:714. doi: 10.1186/s13018-024-05192-y (PMC11531153; doi:10.1186/s13018-024-05192-y)

**Appendix 8**


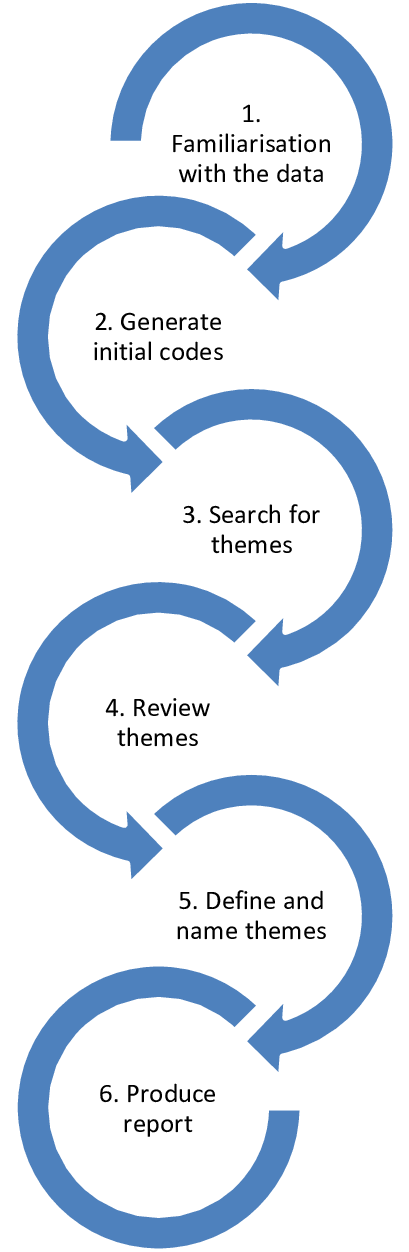


Figure. Six step approach to thematic analysis (Braun & Clarke, 2006)

Supplement: Supplementary file 7 — Supplementary Material 7 [file 13018_2024_5192_MOESM7_ESM.docx]
