# Supplementary material for: Shared decision making in primary malignant bone tumour surgery around the knee in children and young adults: protocol for a prospective study
Source: J Orthop Surg Res. 2024 Nov 2;19:714. doi: 10.1186/s13018-024-05192-y (PMC11531153; doi:10.1186/s13018-024-05192-y)

**Appendix 1**

Images from an animated film that explains the internal prosthesis surgical procedure.


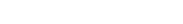

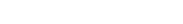

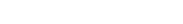

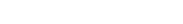

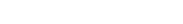

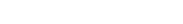

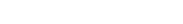

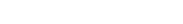

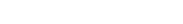

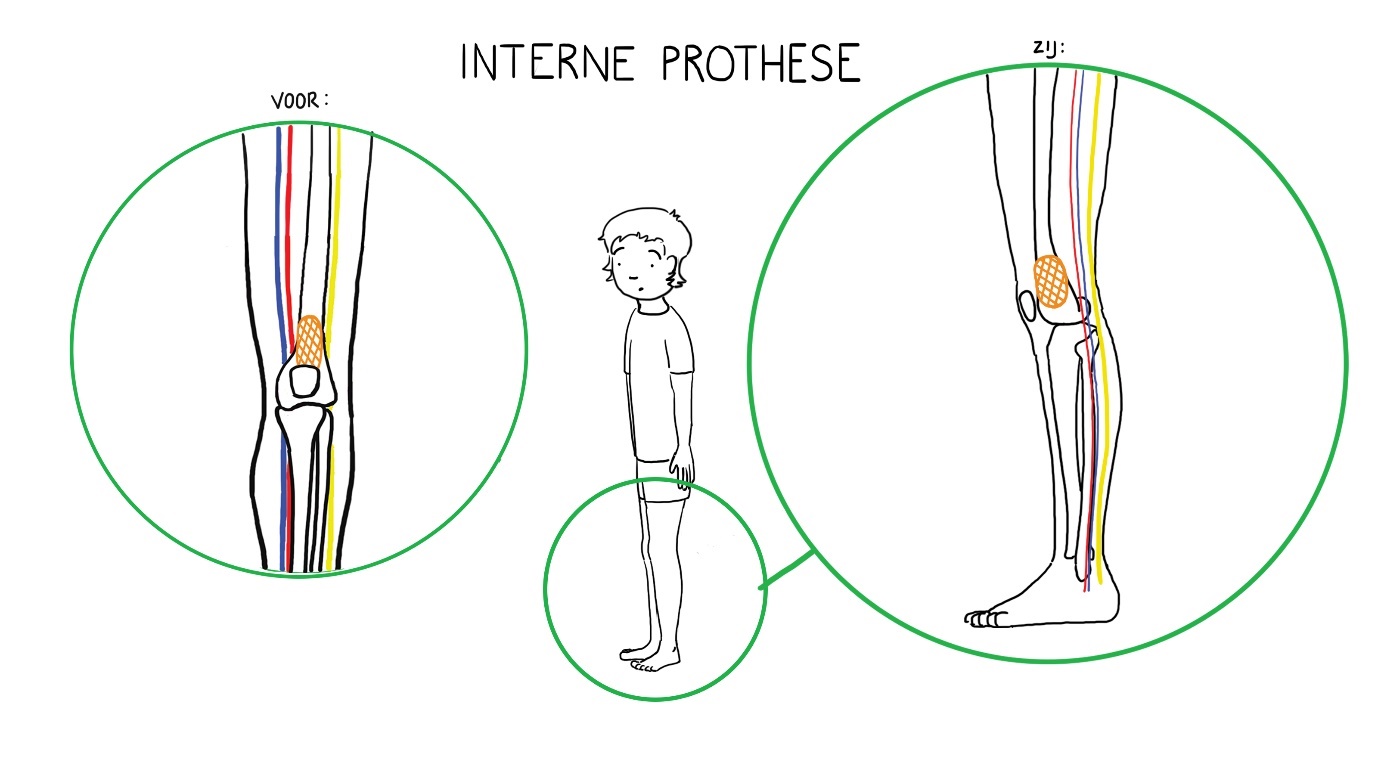

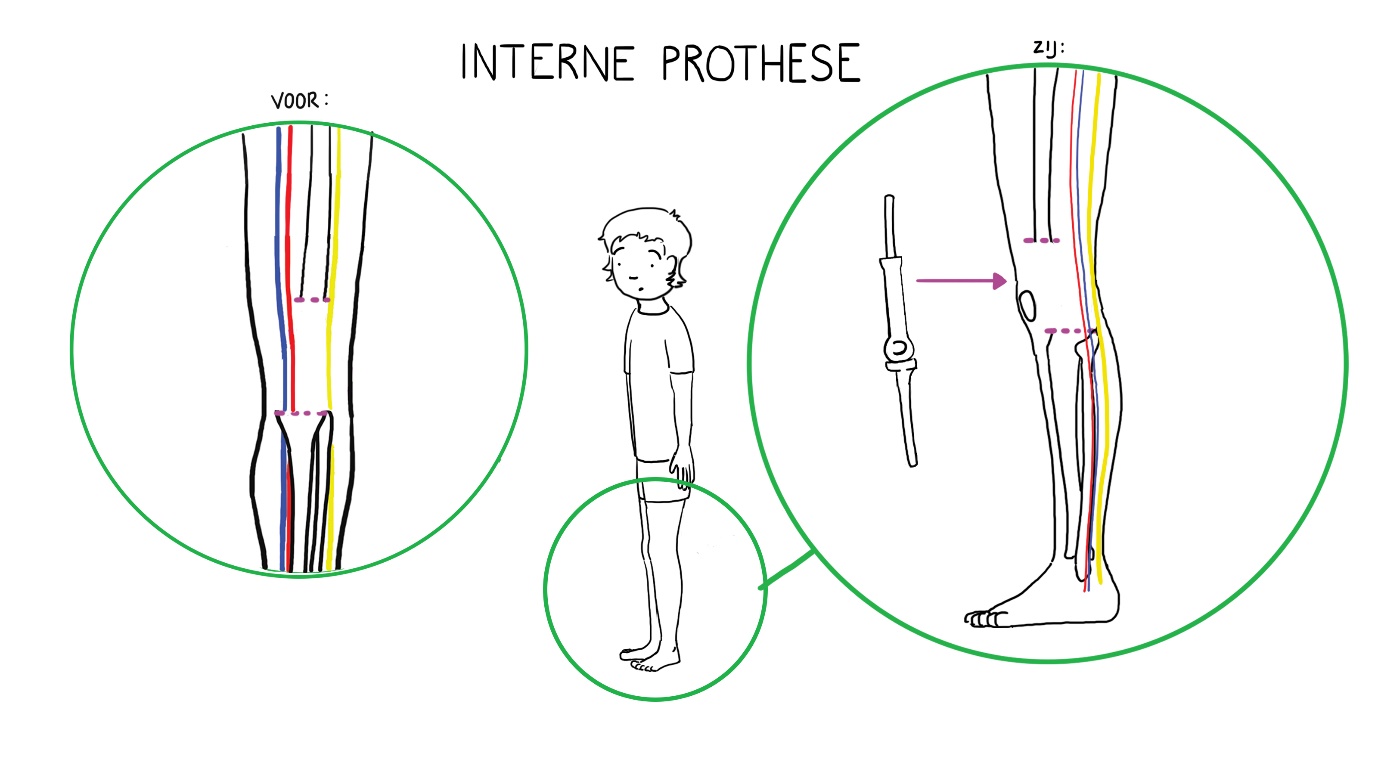

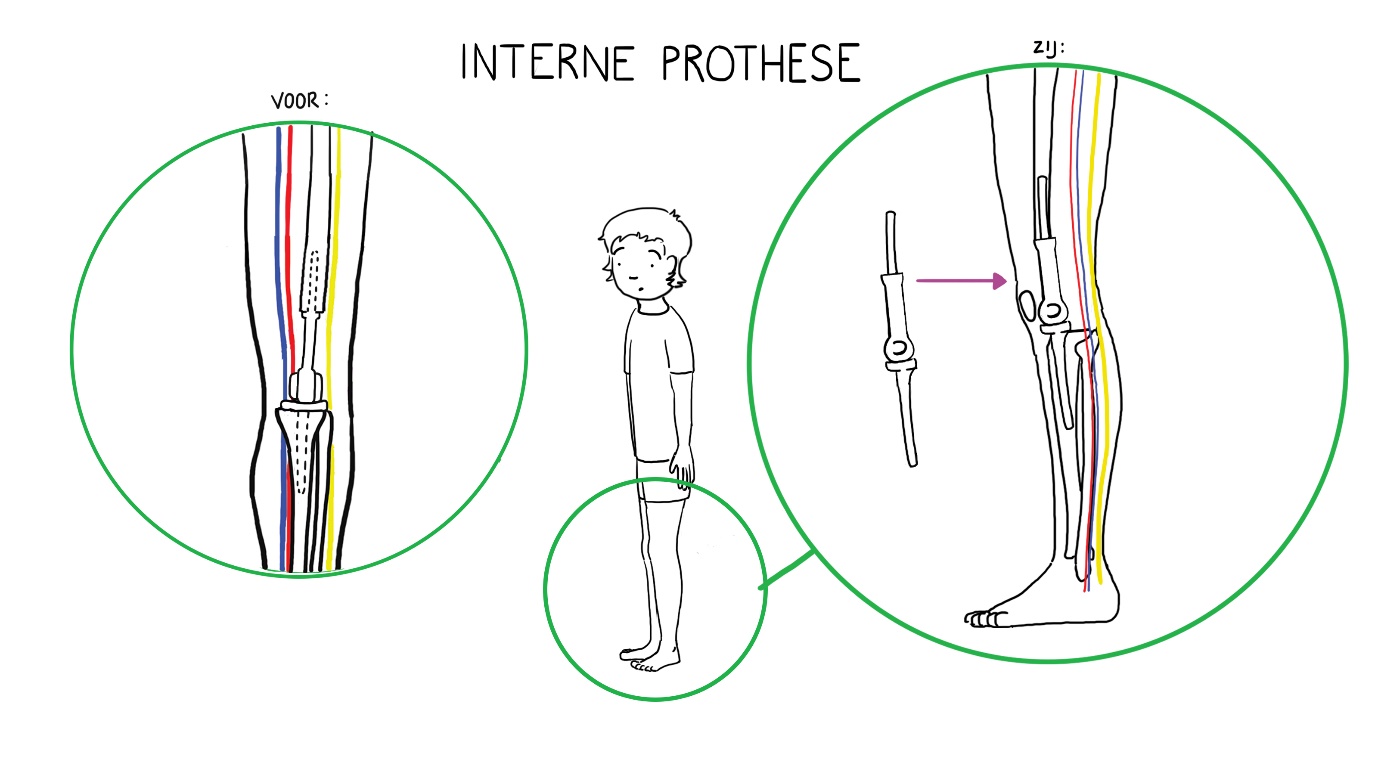


Images from an animated film that explains the tumour resection followed by biological reconstruction surgical procedure.


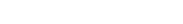

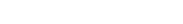

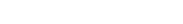

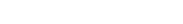

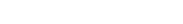

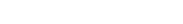

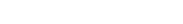

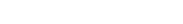

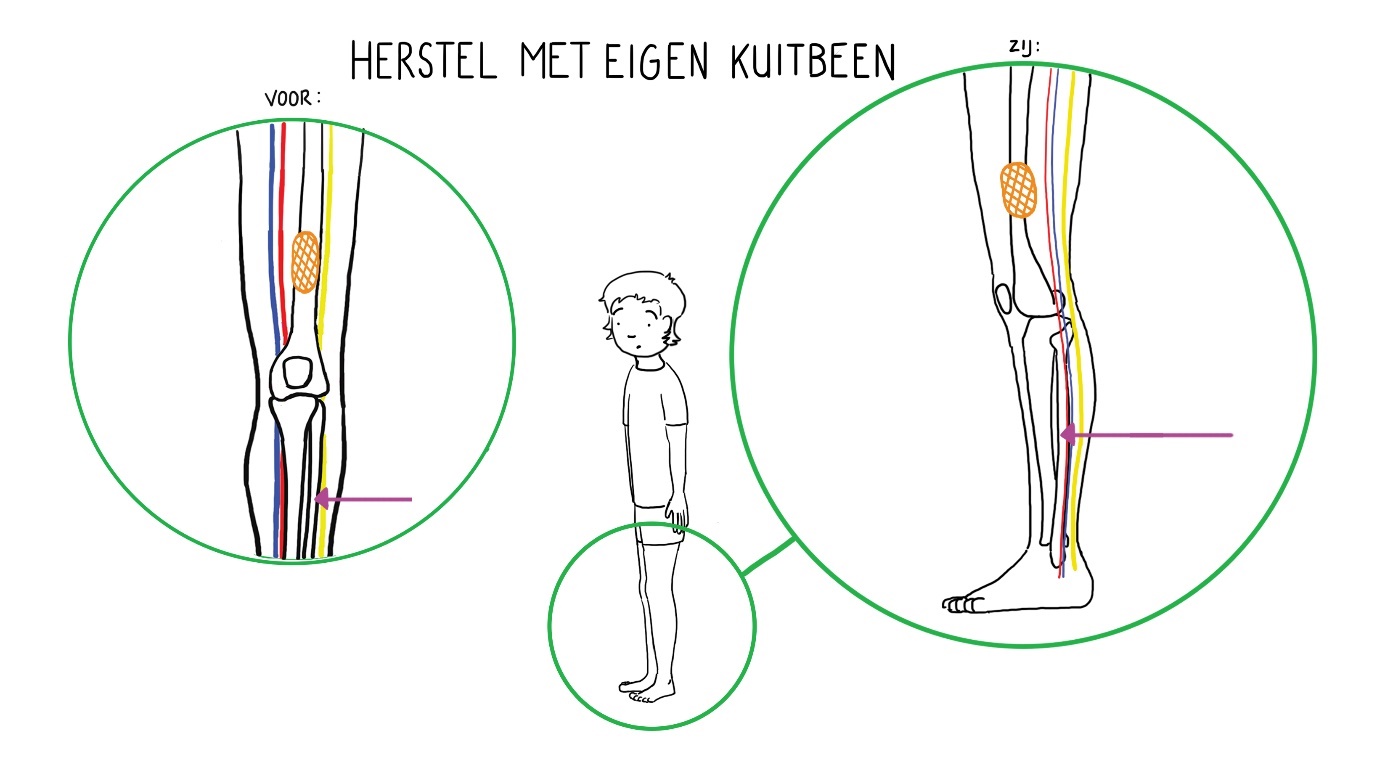

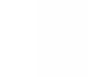

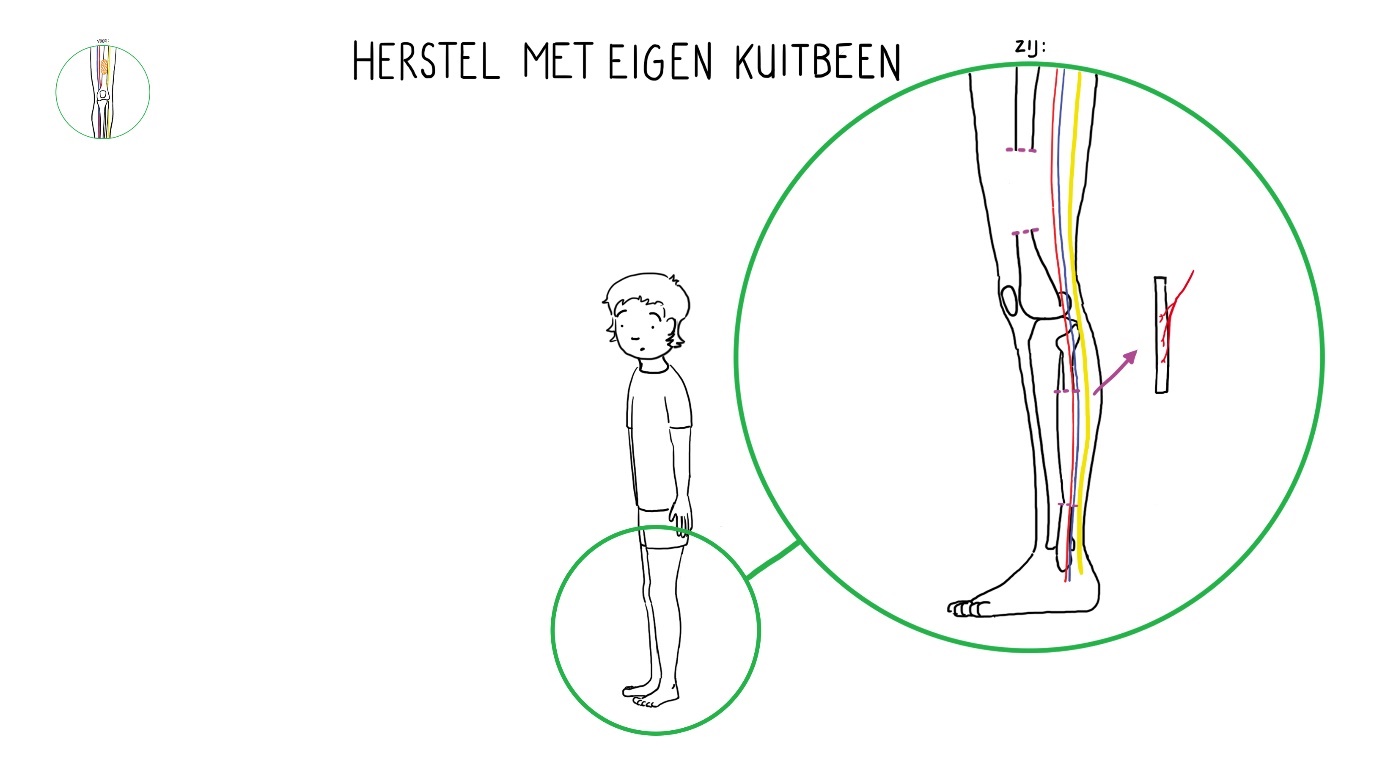

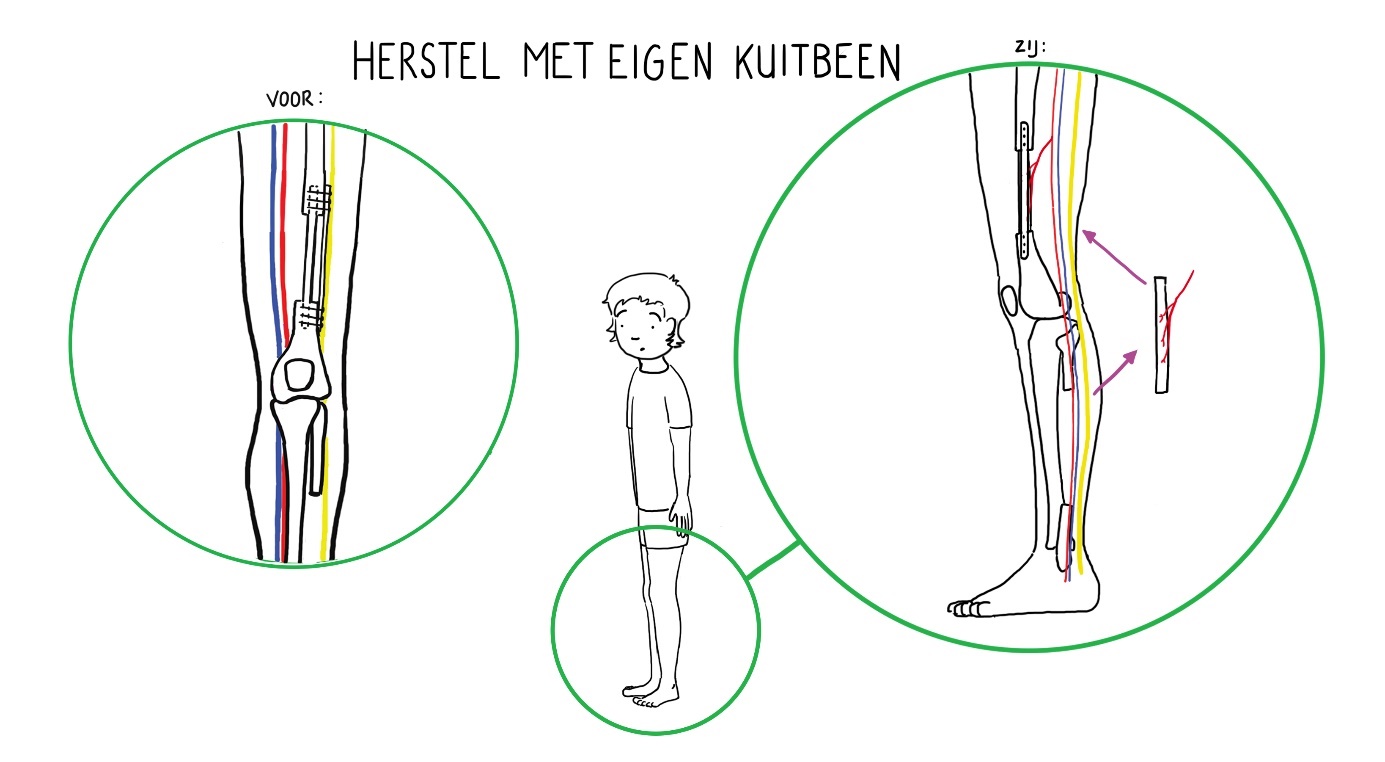
 Images from an animated film explaining the amputation surgical procedure.
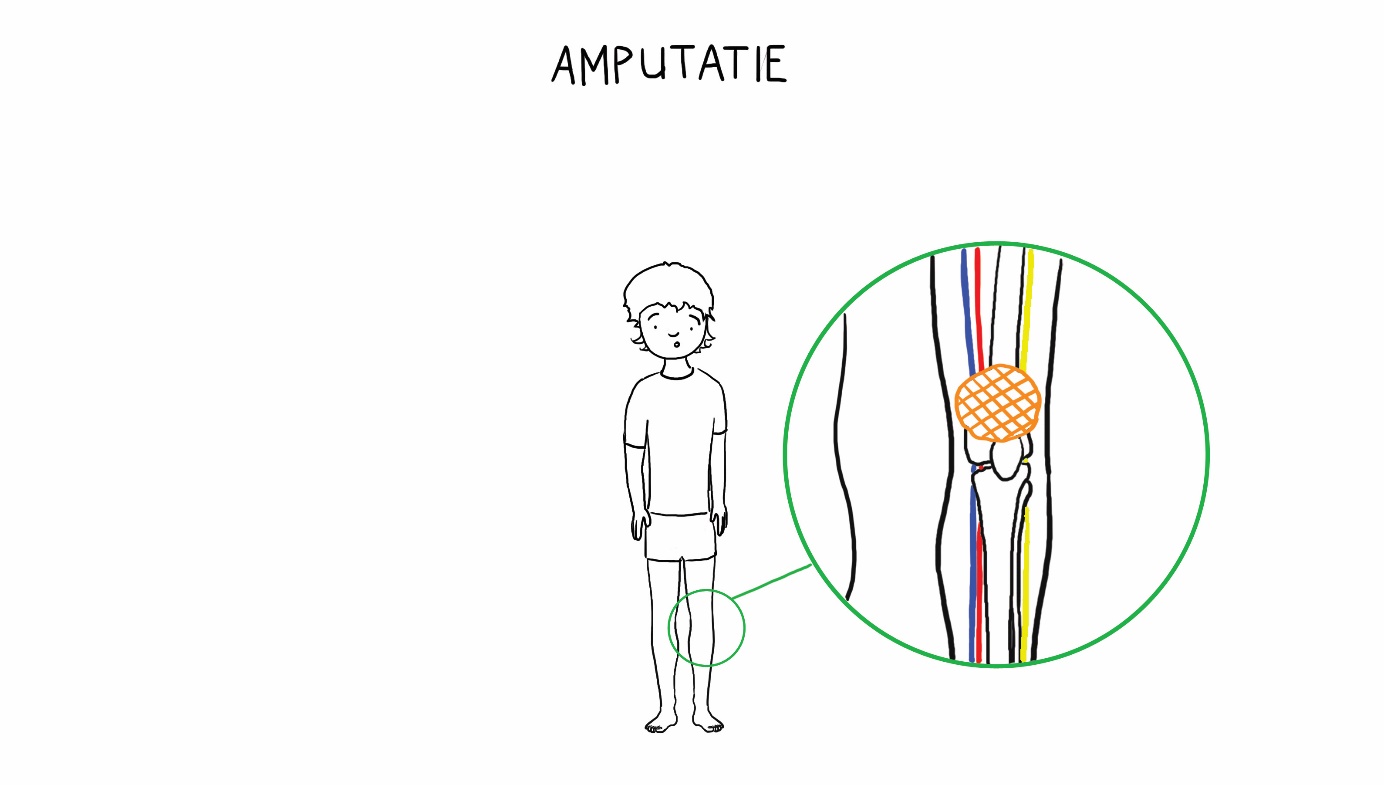


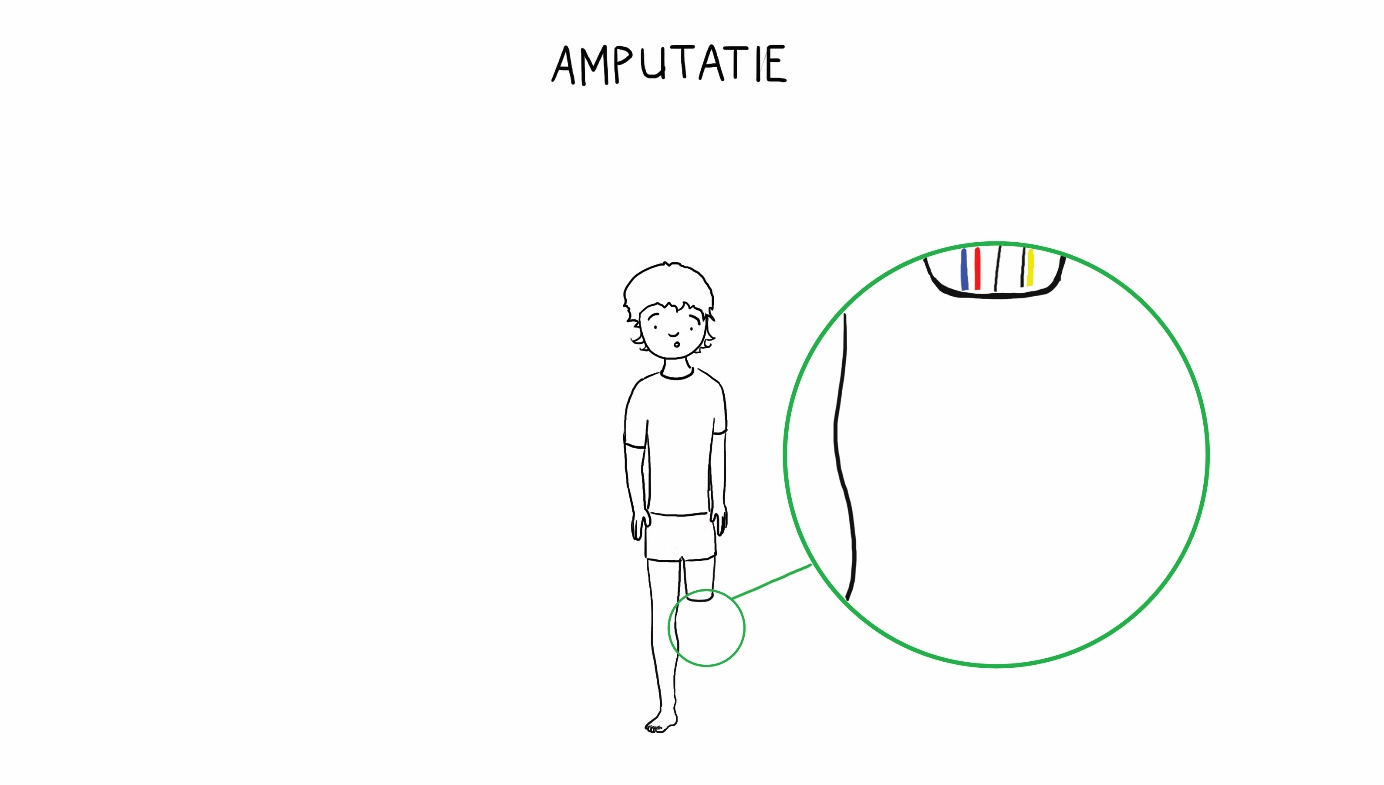


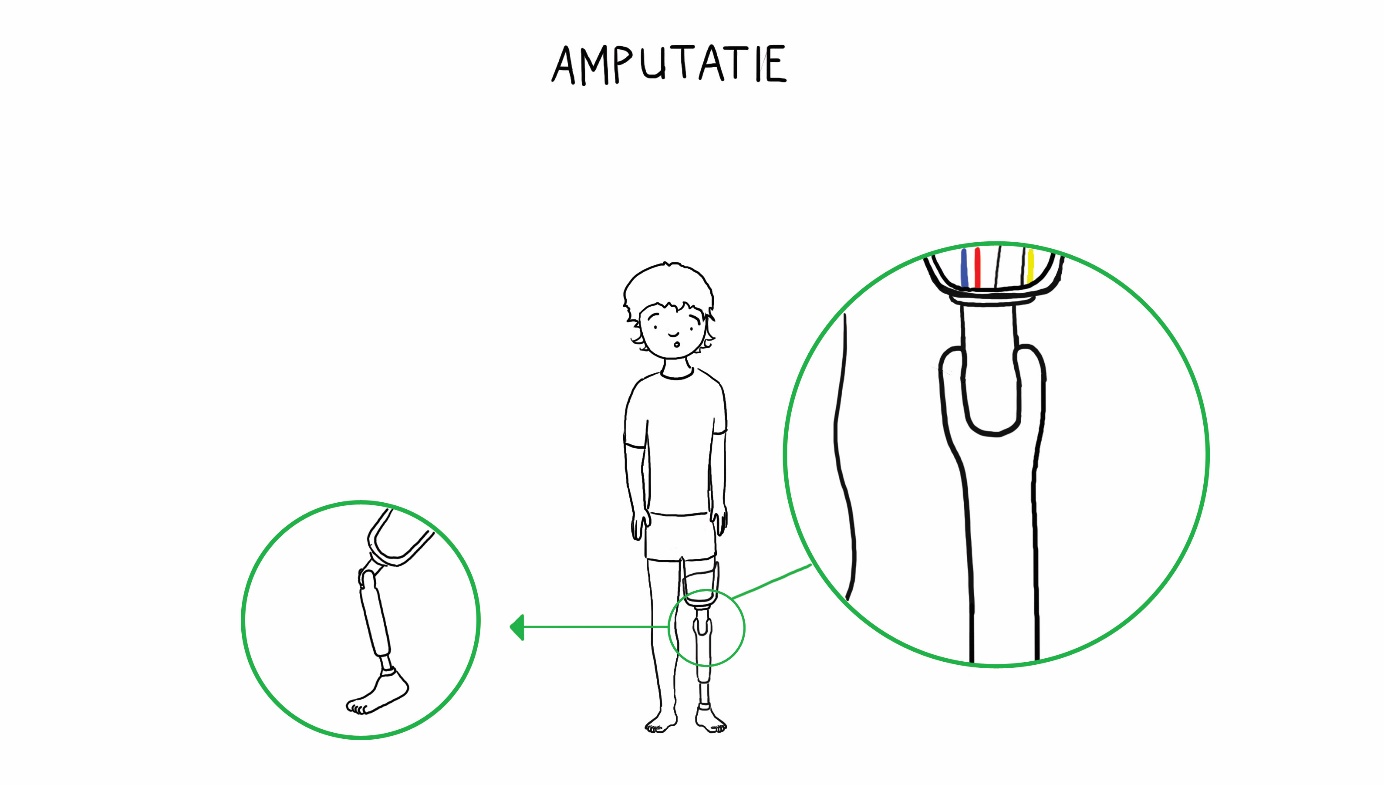
 Images from an animated film explaining the rotationplasty surgical procedure.


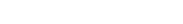

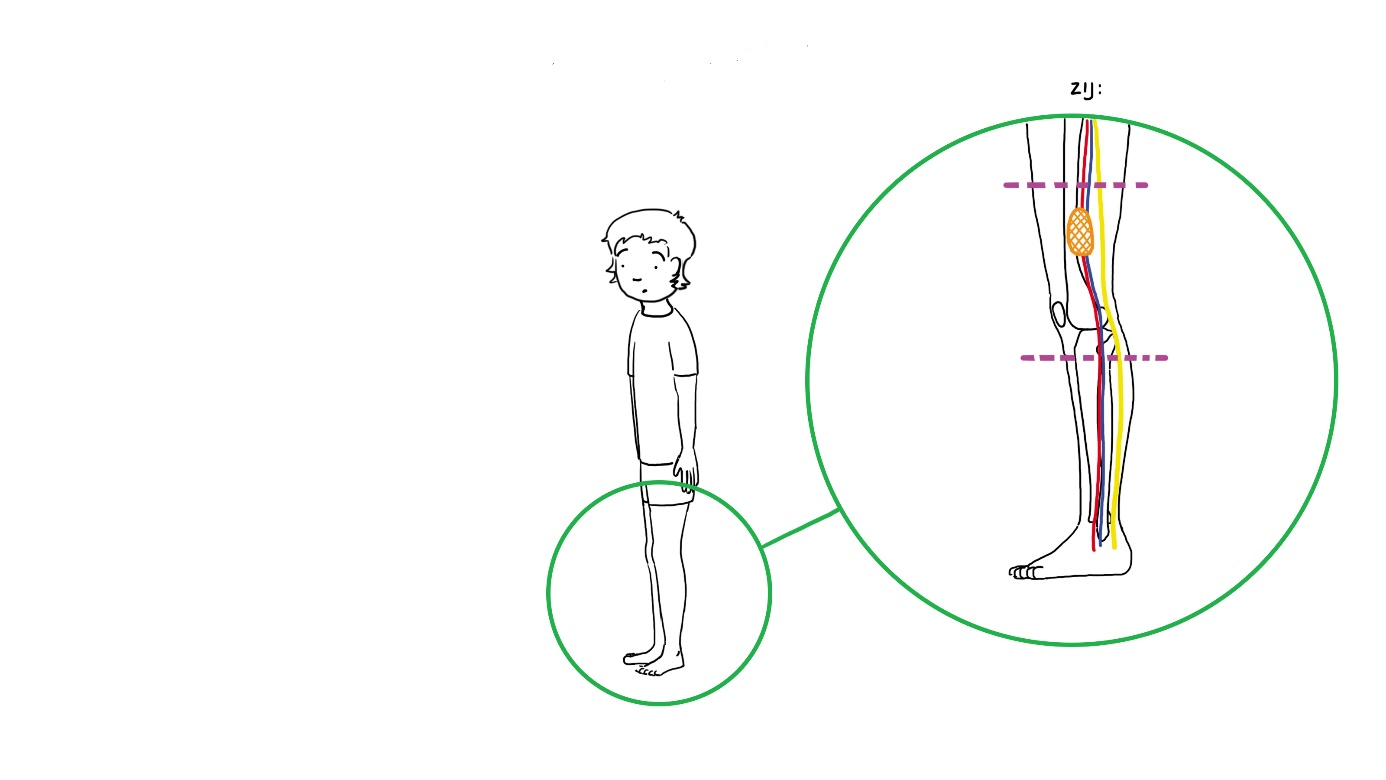


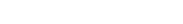

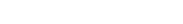

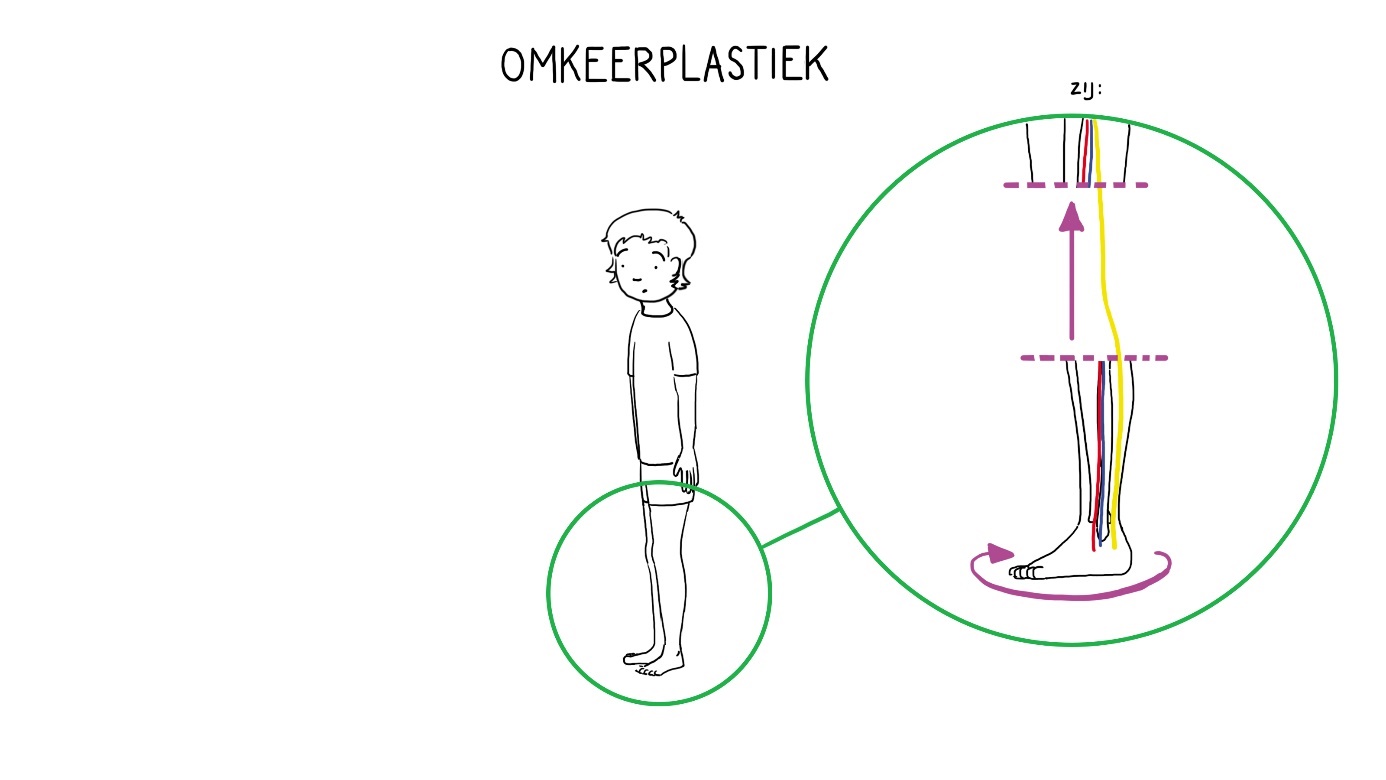


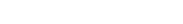

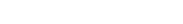

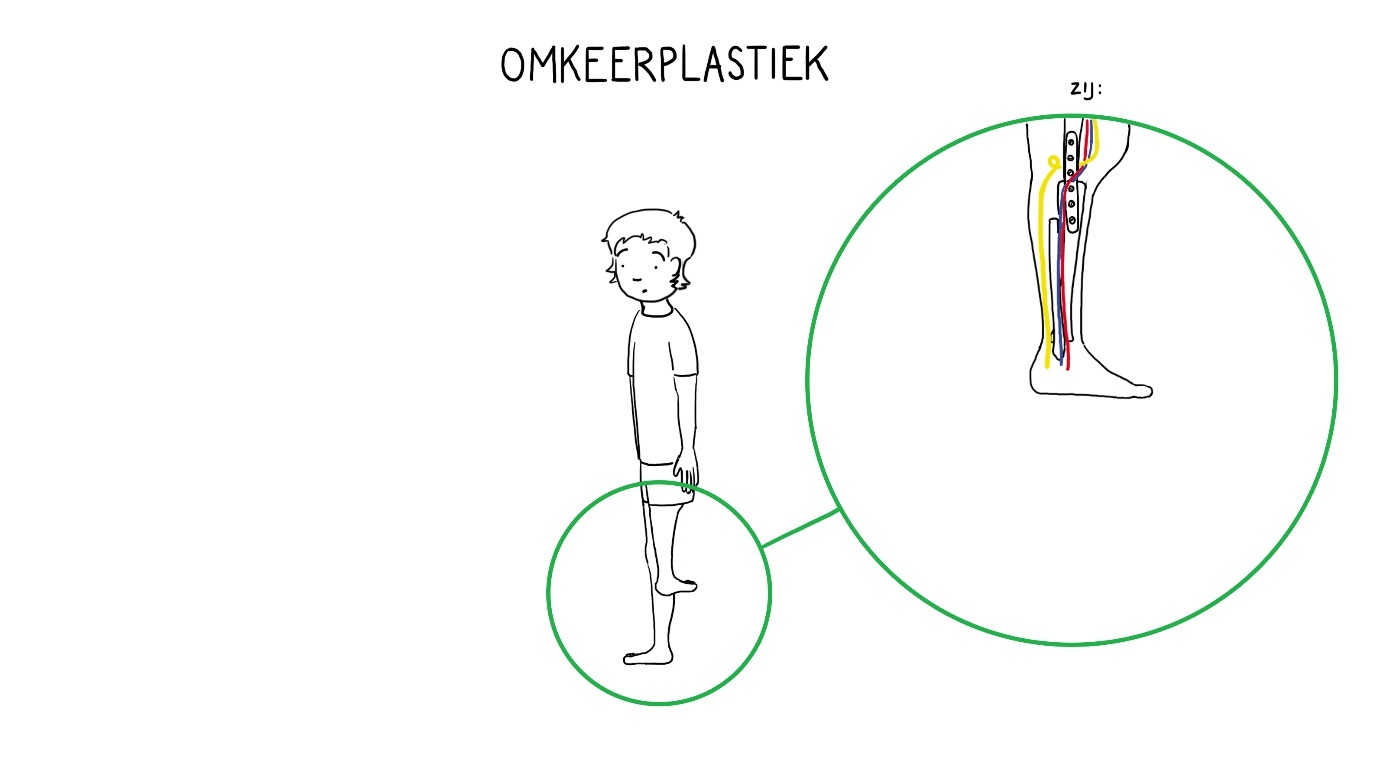

Supplement: Supplementary file 8 — Supplementary Material 8 [file 13018_2024_5192_MOESM8_ESM.docx]
